# Biochemical and Clinical Impact of Organic Uremic Retention Solutes: A Comprehensive Update

**DOI:** 10.3390/toxins10010033

**Published:** 2018-01-08

**Authors:** Raymond Vanholder, Anneleen Pletinck, Eva Schepers, Griet Glorieux

**Affiliations:** Nephrology Section, Department of Internal Medicine, Ghent University Hospital, De Pintelaan 185, 9000 Ghent, Belgium; anneleen.pletinck@ugent.be (A.P.); eva.schepers@ugent.be (E.S.); griet.glorieux@ugent.be (G.G.)

**Keywords:** uremic toxins, uremic toxicity, uremia, Chronic Kidney Disease, CKD, cardiovascular disease, inflammation, fibrosis, patho-physiology CKD, middle molecules, protein bound uremic solutes, water-soluble uremic solutes, This publication contains a comprehensive overview of the current knowledge on uremic toxicity, as well as an estimation of the degree of toxicity attributed to each individual toxin, and a classification according to the degree of known toxicity.

## Abstract

In this narrative review, the biological/biochemical impact (toxicity) of a large array of known individual uremic retention solutes and groups of solutes is summarized. We classified these compounds along their physico-chemical characteristics as small water-soluble compounds or groups, protein bound compounds and middle molecules. All but one solute (glomerulopressin) affected at least one mechanism with the potential to contribute to the uremic syndrome. In general, several mechanisms were influenced for each individual solute or group of solutes, with some impacting up to 7 different biological systems of the 11 considered. The inflammatory, cardio-vascular and fibrogenic systems were those most frequently affected and they are one by one major actors in the high morbidity and mortality of CKD but also the mechanisms that have most frequently been studied. A scoring system was built with the intention to classify the reviewed compounds according to the experimental evidence of their toxicity (number of systems affected) and overall experimental and clinical evidence. Among the highest globally scoring solutes were 3 small water-soluble compounds [asymmetric dimethylarginine (ADMA); trimethylamine-N-oxide (TMAO); uric acid], 6 protein bound compounds or groups of protein bound compounds [advanced glycation end products (AGEs); p-cresyl sulfate; indoxyl sulfate; indole acetic acid; the kynurenines; phenyl acetic acid;] and 3 middle molecules [β_2_-microglobulin; ghrelin; parathyroid hormone). In general, more experimental data were provided for the protein bound molecules but for almost half of them clinical evidence was missing in spite of robust experimental data. The picture emanating is one of a complex disorder, where multiple factors contribute to a multisystem complication profile, so that it seems of not much use to pursue a decrease of concentration of a single compound.

## 1. Introduction

The progressive loss of kidney function in many cases of Chronic Kidney Disease (CKD) is accompanied by the retention of a host of metabolites [1], due to a decrease in their renal clearance that is sometimes accompanied by a rise in generation [2]. Many of these solutes have been shown to exert biological activity, hence affecting the functioning of cells and organs, resulting in the uremic syndrome [3]. The responsible solutes then are called uremic toxins.

At the end of 2016, it was decided to dedicate a special issue of the journal “Toxins” to uremic toxicity. This special issue now comes to an end and contains a number of reviews and original studies. With this narrative review, it is our intention to consider the whole spectrum of uremic toxicity. Such comprehensive review has to the best of our knowledge not been published lately with the last of such initiatives going back to almost 20 years ago [4]. As in the meanwhile much novel data has been generated, we thought it timely to collect this information again in one single publication.

**Figure 1:**
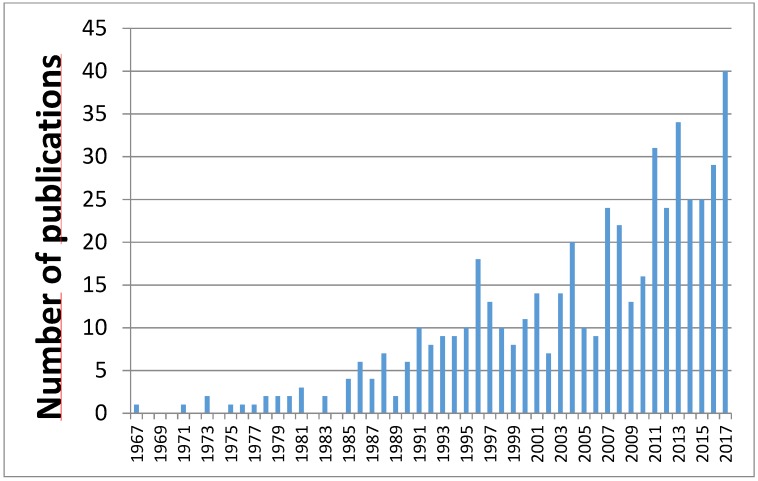
Number of publications per year of appearance included in the reference list of this article.

The knowledge on the identity and the toxicity of the uremic toxins as well as their removal has grown exponentially in the last few years. Analysis of all publications considered for this review shows a rapid year by year increase in number (Figure 1). An encyclopedic listing of known uremic solutes in 2003 identified 90 different compounds [1] and another 56 were added when this effort was repeated in 2012 [5].

The recent acquisitions of metabolomic and proteomic research are enabling to further extend this list [6,7,8,9,10,11,12,13,14,15]. In spite of this almost unlimited possibility for identification it is more difficult and labor intensive to prove the toxicity of the newly detected molecules [6,7]. Hence, it is impossible to discuss all uremic retention solutes that are known today and this review is restricted to the most important ones, either because of their biologic effect or because of their association with clinical outcomes relevant to uremia.

To distinguish among uremic toxins, generally the classification into three major groups as proposed by the European Uremic Toxin Work Group (EUTox) and based on their removal pattern by dialysis is applied [1,16]. It distinguishes between: (1) small water-soluble compounds; (2) protein bound compounds; (3) the so-called middle molecules, which are mostly small peptides (Table 1). Although one could imagine other classification systems, e.g., based on the site of generation, e.g., intestinal or not [17], the currently used approach is preferred because, with the exception of transplantation, extracorporeal dialysis strategies unequivocally remain the most efficient interventional options to decrease the concentrations of these molecules. Such subdivision is in part artificial as in fact there is a continuum in degree of protein binding as well as of molecular weight of uremic solutes. For some (e.g., asymmetric dimethylarginine—ADMA), the degree of protein binding remains a matter of debate [18], whereas for others like p-cresyl glucuronide it is variable and so limited, that not much difference in removal pattern can be expected versus no protein binding [19].

For preparing this review, a list of all toxins worth discussing was prepared (Table 2) and then a literature search via PubMed was undertaken with as search terms the name of the toxin as mentioned in Table 2 on one hand AND toxin/toxicity, uraemic/uremic, CKD or biology, and the literature list was completed with references from previous review papers. Some toxins (e.g., the guanidines) were considered as a group, if in literature they were regularly discussed together. All relevant publications were retained, which implies that some of these point to no toxic effect or even a benefit. Whether the applied concentrations corresponded to those in uremia was not taken into account, as this implies a systematic review process [20]. This review was limited to organic solutes and will thus not deal with inorganic molecules (water, sodium, potassium, phosphorus). 

We also constructed comprehensive tables with the most important biological effects and attributed per toxin a score for the evidence of toxicity (Table 3), whereby arguments in favor of toxicity from experimental or observational studies and randomized controlled trials were taken into account, with a counterbalance if there were also arguments for a neutral or beneficial effect. The scoring system was designed before the literature search was started to avoid bias and to preserve objectivity.

It is acknowledged that the highest clinical level of evidence is obtained from randomized controlled trials (RCTs). However, for most of the toxins considered in this review, randomized controlled trials to check their toxicity are impossible because there are no methods to selectively decrease their concentration. In addition, for the few toxins for which selective concentration decrease is possible (uric acid, homocysteine, Advanced Glycation End products), the few RCTs gave sometimes ambiguous results. Therefore, it was decided not to consider RCTs separately in our scoring system but to account for them together with the observational studies under the common denominator of “clinical studies.”

The scores for number of affected systems and evidence level were compared per uremic toxin group using a non-parametric one-way ANOVA followed by a parametric unpaired *t*-test (GraphPadPrism 5.01 Software, San Diego, CA, USA). *p* < 0.05 was considered as significant. 

## 2. Small Water-Soluble Compounds

The upper molecular weight limit for small water-soluble compounds has arbitrarily been defined as 500 Dalton (Da) (Table 1) and according to their definition protein binding should be minimal. Complete lack of protein binding cannot be excluded but in general should be negligible so as to have no influence on their removal by dialysis, which is efficient with any type of dialysis, although in some cases hampered by kinetic characteristics [21]. Although this removal is dependent on dialyzer blood and dialysate flow and surface area, those characteristics cannot be increased indefinitely to enhance adequacy and therefore probably other approaches such as longer or more frequent dialysis or adsorption should be considered [22].

To the best of our knowledge, the last review on the small water-soluble compounds was published more than 10 years ago [23].

### 2.1. Guanidines

Guanidines are widely spread in nature and a considerable number of compounds is present in humans and retained in uremia [1,5]. The biologic impact of this group of compounds has been demonstrated since many years and the first studies focused on their neurotoxicity, showing their contribution to uremic epiphenomena such as neuroexcitation and impaired cognition, activity and exploratory social behavior [24,25,26,27]. Later studies suggested that the guanidines might also be involved in other uremic complications such as inflammation and inhibition of endothelial cell proliferation, decreased osteoblast calcification and increased osteoclastogenesis [28,29]. Guanidino succinic acid suppresses calcitriol synthesis [30].

Analogous to advanced glycation (see below), guanidines can also promote post-translational protein modification, which has the potential to alter biological function of those proteins [31].

In spite of an origin analogous to that of urea and a molecular weight in the same range, kinetic and removal pattern of the guanidines during dialysis differs significantly from that of urea [21,32].

Asymmetric and Symmetric Dimethylarginine (ADMA and SDMA) are two guanidines with specific biological characteristics [23] that will be discussed separately below.

#### 2.1.1. Asymmetric Dimethylarginine (ADMA)

ADMA has since 1992 been identified as a uremic retention compound with potential hemodynamic impact because it inhibits nitric oxide synthase (NOS), hence decreasing the endothelial protective effect of nitric oxide (NO) [33,34]. Of note, ADMA concentration is increased in a host of settings, many of which are linked to CKD, such as diabetes mellitus, obesity or various forms of cardio-vascular disease [35]. However, it is not always clear whether in the studies identifying high ADMA values in these conditions adjustments for kidney function have been made [35]. ADMA concentration has been linked to cardio-vascular events or mortality in the general population [36,37], as well as in CKD [38,39].

The rise in concentration of ADMA in CKD can to a large extent be attributed to a decreased activity of the enzyme dimethylarginine dimethylaminohydrolase (DDAH), which is responsible for the breakdown of ADMA, rather than to a direct decrease of urinary excretion [18,40].

In healthy volunteers, it was shown that infusing ADMA up to concentrations relevant for uremia had definite hemodynamic effects [41]. Although several attempts have been made to decrease ADMA concentration by drugs or other approaches affecting their metabolism, no indisputable removal method has been identified [35]. Renal denervation in resistant hypertensive patients could decrease ADMA, pointing to the important effect of sympathetic nerve function on its generation [42].

With regards to biologic effects that are of relevance to the uremic condition, ADMA has been linked to polymorphonuclear activation, increased myeloperoxidase release and superoxide production [40,43], expression of adhesion molecules [29] and neurologic alterations such as depression and cognitive disturbances [43,44]. Observational clinical studies as well as experimental studies in wild type CKD mice and in transgenic mice overexpressing DDAH in spite of CKD, suggested also a role in erythropoietin resistance [45]. ADMA levels were also strongly correlated to fibrinogen levels, even at multivariate analysis [38]. Finally, overexpression of DDAH also protected against coronary artery disease in mice with a cardiac graft [46].

#### 2.1.2. Symmetric Dimethylarginine (SDMA)

SDMA is an isomer of ADMA [34] that for a long time has been considered biologically inert [33]. However, during the last decade a number of studies showed that also SDMA was biologically active [18]. First, in endothelial cells, SDMA appeared to decrease NO production, be it via a competition for the NOS substrate, L-arginine, whereas reactive oxygen species (ROS) production was increased [47,48]. In a comprehensive analysis of toxic effects, SDMA appeared to induce inflammation via a broad array of mechanisms essentially affecting leukocytes [29]. Finally, SDMA was detected in uremic High Density Lipoprotein (HDL), transforming HDL into an abnormal lipoprotein inducing endothelial damage [49].

On the clinical level, SDMA was more significantly correlated to serum inflammatory markers than ADMA [48] and it also correlated to mortality and cardio-vascular events in a post-hoc analysis of the Hemodialysis (HEMO)-study, be it to a lesser extent than ADMA did [39]. In the general population, SDMA was associated with mortality, even after adjustment for multiple cardiovascular risk factors, including CKD [50,51]. In the latter two studies, SDMA was a stronger predictor than ADMA [50,51]. Like ADMA, also SDMA could be decreased by renal denervation [42].

In spite of the structural analogies with ADMA, SDMA is not dependent on DDAH for its clearance from the body [34] as it is largely removed by renal excretion [18], with a highly significant correlation with eGFR [47].

### 2.2. Oxalate

Next to the severe oxalate depositions in genetically conditioned primary hyperoxaluria [52], also kidney failure of any cause is associated with tissue depositions of oxalate [53], which can be attributed to supersaturation of serum with oxalate, especially in advanced CKD and dialyzed patients [54]. Very few studies have however assessed the biological impact of oxalate at uremic concentrations. In endothelial cells, oxalate resulted in an increase of intracellular calcium [55], leading to disturbed endothelial proliferation and repair [55,56]. Oxalate also decreases glucuronidation, a detoxifying conjugation process [57].

### 2.3. Phenylacetylglutamate

Phenylacetylglutamate has since long been recognized as a uremic retention product [58] generated by the intestinal microbiota [8]. In recent observational studies the compound has been associated to clinical outcomes relevant to uremia and CKD, such as first cardiovascular event in hemodialysis patients [59], overall mortality and cardio-vascular disease in CKD stages 1–5 [60] and cognitive dysfunction in patients receiving dialysis [61]. To the best of our knowledge, however, it has not been shown whether this compound exerts any direct biological impact. Under certain conditions, it even seems beneficial and an anti-proliferative effect on cancer cells has been demonstrated [62]. Thus, whether phenylacetylglutamate is a real uremic toxin or merely a marker of retention remains for the time being unresolved.

### 2.4. Methylamines

The methylamines (monomethylamine—MMA, dimethylamine—DMA and trimethylamine—TMA) are since long known as uremic retention solutes and may be held responsible for some aspects of malodorous uremic breath [63]. Experimental data on the biological effect of these compounds are very scanty. Preliminary data suggested at least for trimethylamine a link to neurobehavioral disturbances [64]. Methylamine turnover has been associated to vulnerability to atherosclerosis in mice [65]. Dimethylamine and trimethylamine are inhibitors of the organic cation transporter 2 (OCT2), which plays a role in the renal secretion of drugs [66].

In an observational outcome study in hemodialysis patients, methylamine and dimethylamine were however not related to all-cause mortality [67]. On the other hand, monomethylamine has been associated with olfactory dysfunction in hemodialysis patients [68].

Also, trimethylamine-N-Oxide (TMAO) was known as a component of uremic biological fluids [69] already before a boost in studies on this solute occurred from 2011 on when Wang et al. suggested a substantial link to cardio-vascular disease [70]. Its association to CKD and its patho-physiologic role based on experimental and clinical data, are discussed extensively in an article of this special issue [71].

**Figure 2:**
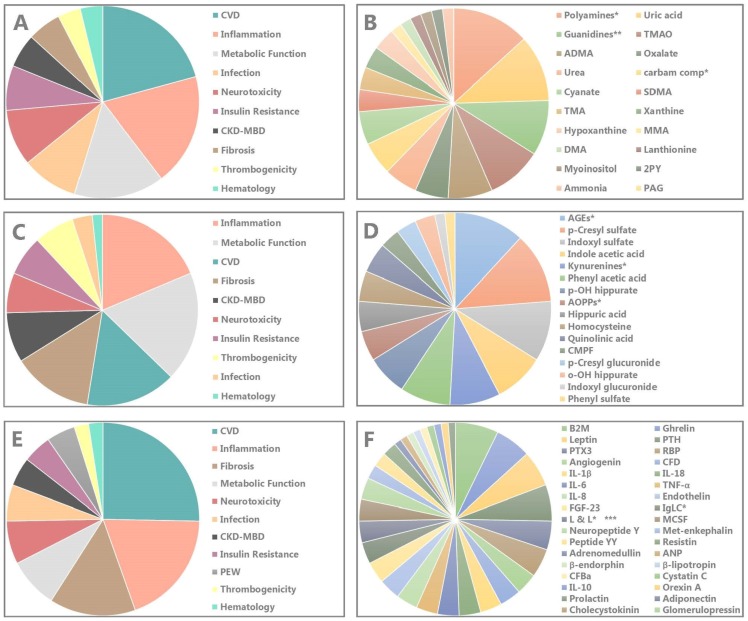
Number of toxins within a group affecting specific systems (left—panels (**A**,**C**,**E**)) and number of systems affected by each individual toxin (right, panels (**B**,**D**,**F**). Small water-soluble compounds (above, panels (**A**,**B**)), protein bound compounds (middle—panels (**C**,**D**) and middle molecules (below—panels (**E**,**F**)). The systems and solutes are ranked clockwise from 12 h on, in proportion to the number involved, the largest numbers coming first. Abbreviations—CVD: cardiovascular disease; CKD-MBD: chronic kidney disease-metabolic bone disease; PEW: protein energy wasting; ADMA: Asymmetric Dimethylarginine; TMA: Trimethylamine; DMA: Dimethylamine; TMAO: Trimethylamine-N-Oxide; carbam comp: carbamylated compounds; SDMA: Symmetric Dimethylarginine; MMA: Monomethylamine; 2PY: N-Methyl-2-Pyridone-carboxamide; PAG: Phenylacetylglutamine; AGEs: Advanced Glycation End Products, p-OH hippurate: p-hydroxyhippurate; AOPPs: Advanced Oxidation protein Products; CMPF: Carboxy Methyl Propyl Furanpropionic Acid; o-OH hippurate: o-hydroxyhippurate; β2M: β_2_-microglobulin; PTX-3: pentraxin-3; IL-1β: Interleukin-1β; IL-6: interleukin-6; FGF-23: Fibroblast Growth Factor-23; L & L: lipids and lipoproteins; CFBa: Complement Factor Ba; IL-10: Interleukin-10; PTH: Parathyroid Hormone; RBP: Retinol Binding Protein; CFD: Complement Factor D; IL-18: Interleukin-18; TNF-α: Tumor Necrosis Factor-α; IgLC: Immunoglobulin Light Chains; Met-Enkephalin: Methionine-Enkephalin; MCSF: Macrophage Colony Stimulating Factor; ANP: Atrial Natriuretic¨Peptide.; * polyamines, carbamylated compounds, AGEs, AOPPs, kynurenines, Immunoglobulin Light Chains and lipids and lipoproteins (modified) considered as one group; ** guanidines considered as one group with the exception of ADMA and SDMA ; *** modified—post-translational.

Intriguingly, in spite of the substantial number of data supporting the toxicity of TMAO [71] (Figure 2B, Supplementary Materials Table S1), the compound is also known as a stabilizer of protein structures [72]. Equally paradoxical, fish as a notorious source of TMAO is protective to cardiovascular disease [73].

### 2.5. Sulfur-Containing Compounds

Whereas a significantly increased exhalation of organic sulfated gases has been observed in uremia [74], a deficiency of the inorganic gas H_2_S also has been observed [75]. H_2_S is generally considered to be vasculoprotective [76,77], thus its deficiency may contribute to uremic vascular disease [78]. Homocysteine is a sulfur-containing uremic retention solute that will be discussed below in the section on protein-bound solutes. Also, lanthionine is a sulfur-containing compound with probably no substantial protein-binding which has been shown to inhibit H_2_S production [75]. Lanthionine is discussed extensively in one of the reviews of this special issue [79]. 

### 2.6. Myoinositol

Myoinositol concentration is increased in cauda equina of uremic patients as compared to healthy controls [80]. Sciatic nerve conduction velocity was decreased in rats treated with myoinositol [81]. Myoinositol also inhibits the proliferation of Schwann cells [80]. In spite of these early data suggesting a role in uremic neurotoxicity, subsequent direct assessments of the impact of myoinositol on nervous function could not confirm this effect [25,82] and some studies even suggest a neuroprotective effect [83].

### 2.7. N-Methyl-2-Pyridone-5-Carboxamide (2PY)

2PY has since long been recognized as a uremic retention solute [1] with a potential of toxicity by inhibition of poly(ADP-ribose) polymerase-1 (PARP-1), an effect possibly impairing DNA replication and repair [84,85]. The interest in this compound was recently boosted by its manifold increase in concentration in CKD patients receiving nicotinamide to attenuate hyperphosphatemia [86]. In a comparative study of nicotinamide with sevelamer, drop-out due to adverse events was 60% higher with nicotinamide, whereby it has been hypothesized that this effect could in part have been due to 2PY [86]. All relevant aspects regarding this compound are elegantly reviewed in this special issue [85] and its experimental and clinical impact is further summarized in Table 4 and Supplemental Table S1.

### 2.8. Polyamines

Although the toxicity of the polyamines has been studied extensively, especially at the end of previous century, interest has somewhat waned during more recent years.

Several structurally related polyamines such as spermine, spermidine, putrescine and cadaverine, may be retained in uremia [1]. Most experimental toxicity data (summarized below) have been accumulated with spermine and spermidine. On the other hand, it has been hypothesized that the previously argued toxicity of especially spermine may be attributed to its oxidative product, acrolein [87] that appears in uremia and uremic samples at the expense of spermine by the activity of the enzyme amine oxidase [88,89]. Recent data indeed show a decrease rather than an increase of concentration in CKD, especially for spermine and spermidine [5,88]. Some of these polyamines may be conjugated to proteins [90].

One of the first biological effects of this group of solutes to be described in the context of uremia, was an inhibition of erythropoiesis [91,92,93]. Polyamine-protein conjugates accumulate in plasma of hemodialysis patients and reduce erythropoiesis via inhibition of mononuclear bone marrow cell proliferation [90]. Spermine also inhibited NOS induction in activated macrophages, which may contribute to deficient immune response [94], while it induced oxidative stress in smooth muscle cells, reducing intracellular glutathione and impairing glucose metabolism [95]. Several polyamines inhibited platelet aggregation [96].

Neurotoxicity of polyamines has repeatedly been assessed. In the earliest studies, this effect was attributed to interference with the *N*-Methyl-D-aspartate (NMDA) receptor which plays a crucial role in channel conductance, leading to neuronal death and neuroexcitation [25,97,98,99]. However, polyamines also play a role in neurotoxicity via other mechanisms, such as NMDA-receptor independent death of neuronal cells [100,101] or interaction with calcium channels [25].

Putrescine inhibits in vitro cell growth of fibroblast-like cells and alters their mitochondrial, cytoplasmic and nuclear membrane structures [102]. Spermine cytotoxicity has also been linked to changes in mitochondrial calcium transport in non-neuronal tissue types such as liver, pancreas and heart [103,104]. The effect on pancreas may play a role in decreasing insulin sensitivity [104]. Finally, in immortalized renal tubular cells several of the polyamines inhibited activity of OCT expression [105].

Of note, some studies also showed beneficial effects, such as improvement of ganglion cell survival and of optic nerve regeneration [106], neuroprotection in brain damage [107] and free radical scavenging [108]. Although those studies in general were not undertaken with uremic toxicity in mind, they might support the hypothesis that the toxicity observed with polyamines is rather generated by metabolites like acrolein [87].

### 2.9. Urea and Derivatives

Urea, a marker of uremic retention in CKD and of adequacy of intra-dialytic solute removal, has traditionally been considered to be biologically inert [109]. However, a number of recent experimental data suggest that urea is toxic at concentrations representative for CKD [110,111,112]. First of all, at least five studies indicate that urea itself directly induces molecular changes related to insulin resistance [113,114], free radical production [113,114,115], apoptosis [116] and disruption of the protective intestinal barrier [117]. Urea also generates cyanate, ammonia and via cyanate carbamylated compounds which as such all have been linked to biological changes (see below). The findings above may increase the patho-physiological underpinning for the validity of KT/V_urea_ as a marker of dialysis adequacy. Yet, also the views that the kinetics of urea are not representative for those of several other uremic retention solutes and that this compound cannot be held responsible for all complex metabolic and clinical changes of the uremic syndrome still remain valid [109].

Increasing urea (and other solute) removal by raising Kt/V_urea_ by more than 30% above standard in an RCT-setting [HEMO-study] could not improve patient outcomes [118] but the results may have been skewed in that study by the short dialysis times [119], which in the Dialysis Outcomes Practice Patterns Study (DOPPS)-analysis predict negative outcomes independently of Kt/V_urea_ [120]. In addition, in a post hoc analysis of the HEMO data, the impact of the rise in Kt/V_urea_ on pre-dialysis concentration of relevant uremic toxins was modest to absent [119,121].

#### 2.9.1. Carbamylated Compounds

The role of carbamylation in CKD has extensively been reviewed elsewhere [122]. Carbamylation is responsible for posttranslational protein modifications resulting in a similar biochemical process as when Advanced Glycation End Products (AGEs) are generated (see below). As they are derived from urea [122], we will discuss them here. Carbamylated compounds are involved in atherogenesis and other functional changes, such as activation of mesangial cells into a profibrogenic prototype, with a potential to play a role in the progression of kidney failure [123] and modification of leukocyte response to collagens [124]. Carbamylated LDL dose dependently induced endothelial cell death and smooth muscle cell proliferation [125] and an increase in monocyte adhesion to endothelium [126], vascular cell changes of relevance to atherogenesis. Also, high density lipoprotein (HDL) is carbamylated in CKD and inhibits endothelial repair function [127].

In at least 3 observational clinical studies focusing on independent hemodialysis populations carbamylated compounds were associated with cardiovascular events and overall and cardiovascular mortality [128,129,130].

#### 2.9.2. Cyanate

Cyanate is a free radical in equilibrium with urea, with increased levels in CKD, as a consequence of the increased availability of urea [131]. Apart from its known link to carbamylation (see above), cyanate also exerts toxicity by itself. Incubation of coronary endothelial cells in the presence of cyanate decreased NOS expression and increased thrombogenic tissue factor and plasminogen activator inhibitor-1 expression [132]. Cyanate also dose-dependently decreased glucose sensitive insulin secretion by pancreatic islets, by a mechanism independent of that generated by urea [114].

#### 2.9.3. Ammonia

Urea is converted to ammonia by urease which is expressed by a number of intestinal bacteria [133]. In vitro studies evaluating expression of intestinal epithelial tight junction proteins not only showed their depletion in the presence of urea but also an even more important decrease when urease, inducing ammonia generation, was added to the medium [117]. This mechanism may contribute to systemic inflammation of CKD by allowing endotoxin from the intestinal lumen to enter the blood stream.

### 2.10. Purines

#### 2.10.1. Uric Acid

Uric acid has been associated with calcitriol metabolism and with inhibition of expression on monocytes of CD14, a lipopolysaccharide receptor [134,135,136].

More recently, uric acid has also been linked to pro-inflammatory processes in cells of the vascular wall, such as monocyte chemoattractant protein-1 (MCP-1) expression by vascular smooth muscle cell [137], induction of oxidative stress [138], renin angiotensin system stimulation and smooth muscle cell proliferation [139]. In human umbilical vein endothelial cells (HUVECs), uric acid induces insulin resistance and suppresses the generation of nitric oxide in response to insulin [140]. Genetic disruption of the uric oxidase gene in mice generated severe hyperuricemia and urate nephropathy [141]. In a series of rat experiments, induction of mild hyperuricemia caused systemic and glomerular hypertension, glomerular sclerosis and renal infiltration of macrophages [142]. All those mechanisms may contribute to hypertension, uremic vascular disease, progression of CKD and thus mortality [143].

In a longitudinal clinical analysis, uric acid was associated with CKD progression, especially in proteinuric patients, even after adjustment for confounders [144]. Uric acid concentration has been associated with parameters of vascular stiffness in participants of the Framingham study [145]. In the National Health and Nutrition Survey (NHANES) study, high uric acid was associated with overall and cardiovascular mortality but the association disappeared after correction for kidney function [146]. In a Mendelian randomization study, both uric acid itself as well as a score composed of a number of genetic polymorphisms associated to high uric acid were associated with multiple cardiovascular complications [147]. In a systematic review and meta-analysis of CKD-patients, uric acid levels were significantly associated with mortality, even after adjustment for kidney function [148]. Uric acid has also been associated with the incidence of acute ischemic stroke [149] and mild cognitive dysfunction [150]. However, in an observational analysis of hemodialysis patients from Taiwan included in this special issue, an inverse relation was found between uric acid concentration and mortality [151]. Similarly, in a large hemodialysis patient database, low and not high uric acid was associated to mortality, especially in those patients with low protein intake [152].

The major problem of observational clinical studies on the impact of uric acid is a risk of confounding as a number of factors commonly linked to elevated uric acid (obesity, diabetes, hypertension), are cardiovascular risk factors by themselves. Many studies adjust for several of those risk factors but residual confounding can never be excluded entirely.

Uric acid takes a unique position among the uremic retention solutes because it is one of the only compounds of which the concentration can be decreased selectively by specific medication, such as allopurinol or probenecid. In an observational study in CKD patients, treatment with allopurinol was associated with improved arterial stiffness, even after correction for confounders [153]. In a Japanese section of the DOPPS, allopurinol was associated with lower overall mortality in a subpopulation without previous cardiovascular events [154].

A few RCTs assessing the impact of uric acid lowering drugs on parameters of endothelial function showed no positive effect [155,156]. On the other hand, in a short-term study, allopurinol was shown to decrease blood pressure in young adolescents with newly diagnosed essential hypertension [157]. In another RCT, in CKD, the group treated with allopurinol had a lower number of cardiovascular events and a slower progression of kidney failure but the small sample size of the study was a limitation factor, so this study needs confirmation [158]. A decrease of uric acid by allopurinol was linked to a rise in serum calcitriol [135].

Thus, in spite of substantial arguments for the toxicity of uric acid, not all data are consistent, especially in RCTs applying uric acid decreasing drugs. In addition, it should be taken into account that the main currently available intervention to lower uric acid, allopurinol, has by itself a considerable complication profile, so that studies suggesting a positive effect should rather be considered as proof of concept than as an incentive for treatment, especially in asymptomatic hyperuricemia.

#### 2.10.2. Xanthine and Hypoxanthine

Xanthine and hypoxanthine are two other purines that sporadically have been submitted to toxicity studies. Both compounds have been linked to inhibition of calcitriol synthesis [134,159] and of membrane linked expression of the lipopolysaccharide receptor, CD14, on monocytes [136].

### 2.11. Summary

Small water-soluble uremic retention solutes can no more be considered as irrelevant to the uremic syndrome, with an average number of affected systems in experimental studies of 2.65 ± 1.89. The systems affected by the largest number of toxins are cardiovascular disease (n = 11), inflammation (n = 10) and metabolic function (n = 8) (Figure 2A, Supplementary Materials Table S1), which all are pivotal for clinical condition and survival. The groups of toxins affecting the largest number of systems are the polyamines (n = 7) and the guanidines (n = 5). For the individual compounds these are uric acid (n = 6), TMAO (n = 5) and ADMA (n = 4) (Figure 2B, Supplementary Materials Table S1). However, with regard to some polyamines, there is debate on whether their concentration is increased or not [5] and whether they all are real toxins [87].

The average evidence score is 1.75 ± 1.29 (maximum possible = 4). For this aspect, the list is headed by the carbamylated compounds for the groups (n = 4) and by ADMA and SDMA (n = 4) followed by TMAO and uric acid (n = 3) for the individual compounds (Table 4).

## 3. Protein Bound Compounds

The protein bound uremic toxins are a heterogeneous group of generally small solutes, which, due to their protein binding, are difficult to remove by dialysis. Their protein binding coefficient is in general low although some compounds may be more stable, especially those generated by posttranslational modifications, such as the advanced glycation end products (AGEs). Many of the protein bound solutes are generated by the intestine [160].

For the time being few therapeutic options exist that specifically can decrease the concentration of this group of solutes and one of these, the orally administered intestinal sorbent AST-120 (Kremezin^R^) did not impact progression of CKD in RCTs [161,162]. For some solutes, specific strategies exist to reduce the concentration of only this compound or group of compounds (e.g., folic acid for homocysteine) and those strategies will be discussed specifically in the subheadings devoted to those molecules.

To the best of our knowledge, the last review considering a large array of protein bound uremic toxins was published almost 10 years ago [163]. After that, reviews have been restricted to specific toxins (e.g., indoxyl sulfate, p-cresyl sulfate) [20,164,165], probably due to the overwhelming number of novel research data.

### 3.1. Advanced Glycation End Products (AGEs)

The role of AGEs in CKD has been reviewed extensively elsewhere [166]. AGEs are generated by the reaction of specific amino acids within the protein structure (both complete proteins and protein degradation products) with the carbamyl group of reducing sugars, which is then stabilized by oxidation. AGE compounds and their precursors are numerous and contain *N*-carboxymethyllysine, pentosidine, hydroxyimidazolone, 3-deoxyglucosone, malondialdehyde, pyrraline, glyoxal and methylglyoxal. AGE accumulation has first been identified in diabetes mellitus [167]. Because CKD as a condition is characterized by inflammation, oxidation as well as retention, AGE accumulation is a typical feature of CKD [168], irrespective of diabetic status [169].

AGEs bind to a number of receptors, among which advanced glycation end product-specific receptor (RAGE) plays a central role to impose several deleterious biological effects [170,171]. RAGE expression is increased in many inflammatory conditions [172] and also in CKD [173,174].

AGEs have a negative impact on body functions and overall outcomes via several mechanisms, among which induction of oxidative stress [175], inflammation [176,177,178] and endothelial dysfunction [178,179,180] including quenching of NO [179], are the most prominent. AGEs have also been linked to thrombogenicity [181], kidney fibrosis [182], structural bone damage [183] and neurotoxicity [184].

With all these functional alterations, it is no surprise that on a more global organic level, AGEs are linked to vascular stiffness, damage and calcification [185,186,187].

Studies on the links between AGEs and mortality gave conflicting results, with some studies even showing better outcomes with higher AGE levels [188,189]. Such results may reflect confounding factors such as better nutritional status resulting in both higher AGEs and higher survival, or the index AGE may have been a compound with low biological impact or with lower vital tissue concentration. In contrast, low serum soluble RAGEs, which are actors protecting against AGE activity, have consistently been linked to cardiovascular risk factors and events [190].

Many interventional attempts have been made to reduce AGE levels either by extracorporeal or by drug treatment. Among the removal strategies high efficiency low pro-inflammatory dialysis options generally turned out to be most efficient but were in general not efficient enough to induce substantial decreases in concentration [191,192,193]. AGE-restricted diet may help to reduce their concentration [194]. None of the tested pharmaceutical options has currently emanated in approved therapies for clinical use [166]. One of the most recent acquisitions is glyoxylase-1 induction, which in pilot studies in obese subjects resulted in the breakdown of methylglyoxal, improved glycemic control, an increase in insulin sensitivity and better vascular function [195,196].

### 3.2. Advanced Oxidation Protein Products (AOPPs)

Similar to AGEs, also AOPPs are posttranslational protein modifications (of both complete proteins and protein degradation products) induced by oxidative stress [197,198]. Both AOPPs prepared in vitro and extracted from plasma of hemodialysis patients have been linked to pro-inflammatory mechanisms, such as induction of oxidative metabolism of leukocytes [198] and free radical production by fibroblasts and endothelium in a setting of scleroderma [199].

Although it has been suggested that their concentration progressively increases throughout CKD to reach a maximum in hemodialysis patients [200] and that these concentrations were predictive of subsequent cardiovascular events [201], concentration measurements are confounded by co-registration of other factors with cardiovascular impact such as triglycerides [202]. Yet, the toxic potential of AOPPs in uremia is less a matter of debate. Of note, RAGE not only interact with AGEs but also with AOPPs, suggesting that many oxidant products interact through the same signal pathways [203].

In this special issue, Garibaldi et al. demonstrate that AOPPs generate transition of macrophages into dendritic cells and an increase of their ROS production, which are key mechanisms in atherogenesis [204].

### 3.3. Carboxy-Methyl-Propyl-Furanpropionic Acid (CMPF)

3-carboxy-4-methyl-5-propyl-2-furanpropionic acid (CMPF), a urofuranic fatty acid, is a strongly lipophilic uremic solute and a major inhibitor of drug protein binding [205]. Next to drugs, it also decreases protein binding of natural metabolites such as thyroxine [206] and bilirubin [207], potentially increasing their biologic/toxic impact. This solute inhibits the renal uptake of p-amino hippuric acid (PAH) in rat kidney cortical slices [208] and competes for renal excretion against various drugs, metabolites and endogenously produced organic acids [209]. CMPF also inhibits hepatic glutathione-S-transferase [210], deiodination of T4 by cultured hepatocytes [211], hepatic digoxin clearance [212] and adenosine diphosphate (ADP)-stimulated oxidation of nicotinamide adenine dinucleotide (NADH)-linked substrates in isolated mitochondria [213]. There is a correlation between neurologic abnormalities and the plasma concentration of CMPF [214]. More recently, CMPF has been associated with inhibition of insulin secretion by pancreatic islets [215,216]. However, in vivo, no correlation between CMPF and parameters of glucose intolerance was found in subjects with features of the metabolic syndrome [217].

Since CMPF is virtually 100 percent protein bound, its removal by hemodialysis strategies is nonexistent but CMPF levels may be lowered with peritoneal dialysis [218]. Since this compound, as several other organic acids, is excreted by the renal tubules [219], enhancing excretion via this mechanism may help to reduce their concentration.

### 3.4. Cresols

#### 3.4.1. P-Cresyl Sulfate

The mother compound of the cresols, p-cresol, was for a long time believed to be a major uremic toxin [220] but later on this point of view was shown to be the result of an artifact [221]. According to two almost simultaneous studies, the real major cresols retained in uremia were the conjugates p-cresyl sulfate and p-cresyl glucuronide [222,223]. Soon after this observation, the exploration started of the biological effect of the conjugate with the highest concentration, p-cresyl sulfate. This resulted in extensive data on its toxicity, summarized in a review article in this special issue [164].

#### 3.4.2. P-Cresyl Glucuronide

P-cresyl glucuronide has far less extensively been studied than p-cresyl sulfate. Although its total concentration is markedly lower than that of p-cresyl sulfate, free “active” concentration is similar [221]. In contrast to p-cresyl sulfate, p-cresyl glucuronide as such has no activating impact on white blood cells but, when added to p-cresyl sulfate, it has an additive effect on leukocyte free radical production [19]. P-cresyl glucuronide also inhibits mitochondrial respiration and the activity of renal glucuronyltransferases [57]. In contrast to p-cresyl sulfate, p-cresylglucuronide did however not trigger insulin resistance and metabolic disturbances [224].

In a longitudinal outcome study in a population with CKD and after adjustment for confounders, including eGFR, p-cresyl glucuronide showed a similar correlation to overall and cardiovascular mortality as indoxyl sulfate and p-cresyl sulfate, suggesting a comparable impact on mortality [225].

### 3.5. Hippurates

#### 3.5.1. Hippuric Acid

Hippuric acid is since long known as an inhibitor of drug protein binding [226] but other biological effects have been less extensively explored. The compound has been associated with kidney damage and to proximal tubular injury [227,228,229], an effect related to organic acid transporter uptake [228]. Of note, also several metabolomic studies linked hippuric acid to renal tubular damage [230,231,232]. The only other toxic effects that were described were an inhibition of glucose utilization [233] and a decrease of digoxin uptake by hepatic cells [212].

#### 3.5.2. p-Hydroxyhippuric Acid

p-Hydroxyhippuric acid induced free radical production in renal tubular cells [228], inhibition of tubular cell proliferation [228] and inhibition of Ca^++^-ATPase, possibly contributing to uremic bone disease, disturbed Ca^++^ homeostasis and hypertension [234]. A similar effect of disturbed cellular Ca^++^ efflux had previously been described in uremic plasma filtrate but that study had not been able to unravel the responsible solute(s) [69]. p-Hydroxyhippuric acid also attenuates leukocyte apoptosis, which may result in a pro-inflammatory effect [235].

#### 3.5.3. o-Hydroxyhippuric Acid

Also, o-hydroxyhippuric acid induced free radical production in renal tubular cells and inhibition of tubular cell proliferation [228].

### 3.6. Homocysteine

Homocysteine (Hcy) is a sulphur-containing amino acid produced by demethylation of methionine and a cardiovascular risk factor in the general population. Its concentration is markedly increased in end stage kidney disease (ESKD) [236]. Retention in uremia results in the cellular accumulation of S-adenosyl homocysteine (AdoHcy), an extremely toxic compound that competes with S-adenosyl-methionine (AdoMet) and inhibits methyltransferases [237], a mechanism disturbing epigenetic control of gene expression by provoking DNA hypomethylation [238].

Hcy increases the proliferation of vascular smooth muscle cells, one of the most prominent hallmarks of atherosclerosis [239]. The administration to rats of excess quantities of the Hcy precursor, methionine, induces atherosclerosis-like alterations in the aorta [240]. Hcy also disrupts several vessel wall-related anticoagulant functions, potentially resulting in enhanced thrombogenicity [241].

More recent data have shown that hyperhomocysteinemia also is linked to a decreased concentration of hydrogen sulfide (H_2_S), a volatile vasorelaxant [242] and to the increased generation of lanthionine, a condensate of two homocysteine molecules, which in its turn decreases H_2_S [75,79].

Serum homocysteine levels not only depend on kidney function but also on genetic predisposition [243] and vitamin status [244]. Homocysteine levels can be reduced by the administration of folic acid, vitamin B6 and/or vitamin B12 [245,246]. CKD patients may be refractory to regular folate doses as applied to the general population and may require higher quantities than the non-uremic population.

When analyzing the relation between homocysteine levels and hard outcomes in CKD, two large, observational studies concluded that low rather than high homocysteine levels were associated with poor outcomes [189,247] but potential confounders are the frequent inflammatory and malnourished status of CKD patients. Along this line of thought, one study demonstrated that homocysteine was directly related to mortality in that part of a hemodialysis population that did not suffer from a chronic inflammation-malnutrition state (CISM) [248]. This relationship was masked in patients with CISM [248]. Another carefully conducted analysis, however, found no association between total homocysteine and cardiovascular disease risk [249].

In four RCTs in CKD vascular events or fatal outcomes were not reduced by homocysteine lowering therapies with administration of folic acid alone or combined to other B vitamins [250,251,252,253]. However, in all but one of these studies the control patients had been subjected to folic acid fortification in regular food [250,251,252].

A sub-analysis of a large RCT on the effect of folic acid on stroke in a large population of hypertensive Chinese [254], showed in the subgroup with CKD, that folic acid combined to enalapril more efficiently refrained progression of CKD than enalapril alone [255]. This study obviated the bias of folic acid fortification in the general population but on the other hand was restricted to hypertensive Chinese, which might have implied specific genetic or metabolic factors.

Two meta-analyses suggested a reduction of cardiovascular disease associated with the application of homocysteine lowering preparations in CKD [256,257]. This effect was seen especially in subpopulations that were not or only partially subject to folic acid food fortification, or that showed a decrease of homocysteine by more than 20% in the treated group, irrespective of folic acid fortification [257]. Many studies in these meta-analyses were small, which might have detracted from the credibility of the analysis.

Dialytic removal of homocysteine is thought to be hampered in a similar way as for the other protein bound uremic toxins. Dialysis with a membrane with very large pores could decrease homocysteine concentrations, possibly due to a modification of metabolism, rather than to direct removal [258].

Thus, attempts to study the impact of homocysteine concentration on cardiovascular outcomes emanated in conflicting results but some of these studies might be subjected to confounding. On one hand, observational studies may have been confounded by malnutrition and inflammatory status. On the other, controlled studies with vitamin B preparations may have been confounded by folic acid fortification in the general population. However, clinical data seem too conflicting to offer convincing arguments about the role of homocysteine in CKD, possibly because its impact is, in contrast to the general population, overridden by the toxic effect of other solutes.

### 3.7. Indoles

Several of the indoles, which are tryptophan metabolites, are retained in CKD. Also, quinolinic acid, although not properly considered as an indole, is a tryptophan metabolite (see below).

#### 3.7.1. Indoxyl Sulfate

Indoxyl sulfate has in the context of uremic toxicity definitely been the most extensively studied of all indoles. Although there has been debate whether in those studies the correct concentrations were used [259], a systematic review found a substantial number of studies applying the right conditions [20]. Clinical evidence of toxicity in humans however is only observational and several studies showed no association [165]. This special issue contains an extensive review on the toxicity of indoxyl sulfate [165] with an addition to this review in a letter to the editor [260]. This special issue also contains a publication describing an oxidative effect and inhibition of nitric oxide in endothelial cells by indoxyl sulfate that is counteracted by uric acid [151] and two other studies respectively assessing the role of indoxyl sulfate in inhibiting neovascularization [261] and in enhancing coagulation [262].

#### 3.7.2. Indole Acetic Acid

Indole acetic acid in general received less attention in uremia research than indoxyl sulfate but this trend has changed especially during the last few years. Indole acetic acid has been linked to cardiovascular disease by induction of pro-inflammatory mechanisms [263,264] and pro-coagulant tissue factor production [265]. Like for indoxyl sulfate, these effects to a large extent are linked to the activation of the Aryl Hydrocarbon Receptor (AhR) that seems to play a central role in the biological action of several indoles [266].

Indole acetic acid also induces proximal tubular injury after uptake via organic acid transporters, stimulates cellular free radical production [228] affecting cell viability [229] and accelerates progression of CKD in experimental animal studies [227]. Furthermore, indole acetic acid has been linked to a number of metabolic effects, such as the inhibition of uptake of digoxin [212] and renal glucuronidation [57].

In one study indole acetic acid had no pro-oxidant effect [267] but this compound may need to be oxidized to become biologically active, as suggested by a study in a hematological malignancy setting, showing a cytotoxic effect of oxidized indole acetic acid [268].

In an observational clinical study, indole acetic acid was associated with increased mortality and cardiovascular events [263].

#### 3.7.3. Indoxyl Glucuronide

Although it is known since some years that also indoxyl-β-d- glucuronide is retained in uremia [269], arguments in favor of a biological effect are scanty. Indoxyl glucuronide is one of the uremic solutes inhibiting the OCT2, which plays a role in the renal secretion of drugs [66].

### 3.8. Kynurenines

Elements of the kynurenic pathway are increased in CKD, not only because of decreased renal clearance but also of alterations in their metabolism [270]. They have been shown to interfere with organic functions, such as causing an increase of leukocyte adhesion to vascular endothelium [271], activation of AhR [266] and decreased lipopolysaccharide-induced production of Tumor Necrosis Factor-α (TNF-α) [272]. Injections of kynurenic acid in rats with normal kidney function impaired their cognitive flexibility [273]. Furthermore they also modify metabolic functions by impairment of hepatic drug uptake [274], the activity of renal glucuronyltransferases [57] and energy metabolism by rat cerebral cortex [275].

In the general population, kyurenine levels were associated with all cause, cancer and cardiovascular mortality [276] and in patients with stable angina and myocardial infarction [277]. In patients with ESKD, the kynurenines were associated with oxidative stress, inflammation and prevalence of cardiovascular disease [278] and parameters of hypercoagulability [279].

### 3.9. Phenols

Of the phenols, phenylacetylglutamine, which conceivably is very little bound to protein, has been discussed above, in the section dealing with the small water-soluble compounds.

#### 3.9.1. Phenyl Sulfate

To the best of our knowledge, the toxic effects of phenyl sulfate in the context of uremia have only rarely been studied. The solute affected tubular cell viability [229].

#### 3.9.2. Phenyl Acetic Acid

Phenyl acetic acid as a protein bound uremic toxin does not seem to compete for its protein binding with other major protein bound uremic toxins like p-cresyl sulfate or indoxyl sulfate [280]. Phenyl acetic acid has been linked to inhibition of inducible nitric oxide synthase (iNOS) expression [281], osteoblast proliferation and differentiation [282], enhancement of the formation of oxygen free radicals in vascular smooth muscle cells [283] and increases of inflammatory response by polymorphonuclear leukocytes [284]. On the metabolic level, it inhibits renal cellular solute transport pumps [13] and conjugation by glucuronidation [57]. Finally, in a clinical study, phenyl acetic acid was related to cognitive dysfunction in dialysis patients [61].

### 3.10. Quinolinic Acid

Quinolinic acid is an oxidative product of tryptophan metabolism [285]. Also for this compound concentration may at least in part be increased in CKD by changes in the kynurenine metabolic pathway [270]. It contributes to the inhibition of erythropoiesis [285] and neurotoxicity [286]. The latter effect occurs via the intermediate of neuroinflammation [286].

### 3.11. Summary

Like with the small water-soluble molecules, these data show that also the protein bound solutes have the capacity to affect a host of biological systems that contribute to the uremic syndrome (Figure 2C, Supplementary Materials Table S2), with an average number of experimentally affected systems of 3.69 ± 1.96. The systems affected by the largest number of toxins are inflammation (n = 11), as can be expected with protein bound solutes metabolic function (n = 11) and cardiovascular damage (n = 9). The group of toxins affecting the largest number of systems are the AGEs (n = 7) followed by the kynurenines (n = 5). As far as the individual compounds are concerned the list is headed by p-cresyl sulfate (n = 7), followed by indoxyl sulfate (n = 6), indole acetic acid and phenyl acetic acid (n = 5) (Figure 2D).

The average evidence score is 2.31 ± 0.95 (maximum possible = 4). For this aspect, the list is headed by the groups of the kynurenines (n = 4—the maximum score), followed by the AGEs (n = 3). The highest scoring individual compounds were p-cresyl sulfate (n = 4) and indoxyl sulfate, indole acetic acid and phenyl acetic acid (score of 3) (Table 5).

Although most of the average evidence scores are not significantly different, the experimental data for the protein bound compounds seem to be more robust than the clinical ones. Indeed, toxins like homocysteine and indoxyl sulfate are not covered by unequivocal clinical data, whereas others like p-hydroxy hippuric acid, indole acetic acid, quinolinic acid and phenyl acetic acid have barely been studied clinically in spite of fairly robust experimental data.

## 4. Middle Molecules

The group of the middle molecules is mainly composed of small proteins or peptides that under normal conditions can cross the glomerular filtration barrier (<58,000 Da). Their minimum molecular weight has arbitrarily been set at 500 Da although most middle molecules have a molecular weight of more than 10,000 Da. The group contains both intact molecules and their degradation products. This degradation has been well described for some compounds (e.g., parathyroid hormone) but for many others not.

In contrast to the small water-soluble compounds and the protein bound solutes which to a large extent are intestinal metabolites of nutrition components, most of the middle molecules are generated endogenously. They are often part of homeostatic mechanisms to correct for other uremic changes (e.g., parathyroid hormone), or are expressed in response to other toxins (e.g., the cytokines), whereby their concentration not only depends on retention but also on endocrine and paracrine corrective mechanisms. A typical example is parathyroid hormone of which the concentration changes to a large extent are defined by alterations in bone homeostasis.

Because of their high molecular weight, dialytic removal is only possible with membranes with a large enough pore size (high-flux membranes) that can be used in a diffusive (hemodialysis) or convective mode (hemodiafiltration). To the best of our knowledge, few strategies exist to decrease concentration of specific middle molecules. Increasing their unspecific removal by applying large pore membranes often resulted in conflicting results with a number of clinical studies, even on hemodiafiltration as the most effective strategy, showing no hard outcome advantage at primary analysis [287,288,289,290]. Other studies showed a survival advantage but were prone to selection bias [291].

### 4.1. Adrenomedullin

The concentration of adrenomedullin is increased in dialysis patients but decreases when volume overload is corrected [292], which is suggestive of active secretion in overhydration. The molecule decreases intestinal epithelial permeability [293] and corrects cardiovascular malfunction [294]. It also has a mitogenic effect [295], which could be useful in repair function and for immune response but may also accelerate cell cycle in malignancy. Apart from the latter possibility, there are no manifest reasons to consider adrenomedullin as a real toxin. It rather seems a beneficial molecule with even a potential to correct for a number of elements of the uremic syndrome.

### 4.2. Adiponectin

Adiponectin is one of the adipokines of which the concentration is increased in CKD [1,5]. It is one of the compounds discussed in a review of this special issue on the cytokines [296]. However, the latter publication contains ample arguments stressing the uncertainties regarding the toxicity of this compound.

### 4.3. Angiogenin

Angiogenin is a member of the ribonuclease (RNAse) superfamily that next to stimulating angiogenesis, e.g., in cancer but also in wound repair [297], has been linked as well to inhibition of polymorphonuclear leukocyte degranulation [298], cytotoxicity and inhibition of protein synthesis [299].

### 4.4. Atrial Natriuretic Peptide

Atrial natriuretic peptide (ANP) as well as its sequence homologue, brain natriuretic peptide (BNP), are retained in uremia [1] and are independently linked to left ventricular mass and function and to overall and cardiovascular mortality in dialysis patients [300]. Yet, experimental studies on its physiologic impact not necessarily point to deleterious actions, as some data rather seem to suggest counter regulatory effects neutralizing a number of elements potentially contributing to the uremic syndrome. Enhanced ANP secretion has been suggested to play a role in the adaptive increase of sodium excretion per nephron in CKD [301] and decreases catecholamine release from nerve growth factor-treated rat pheochromocytoma cells by inducing a neuromodulatory effect [302]. When infused in rats with hypoxia-induced pulmonary hypertension, ANP attenuated pulmonary hypertension and reduced pulmonary vascular remodeling [303] and it also has been attributed a blood pressure lowering effect [304]. ANP also increases insulin levels in vivo [305], while in isolated rat pancreatic cells it inhibits glucagon secretion [306]. Both effects have the potential to restore at least in part the disturbed response to insulin in uremia. Maybe the only somewhat negative action we could retrieve is the inhibition of Follicle Stimulating Hormone (FSH) induced maturation of pig oocytes [307]. Of note, N-terminal pro-B-type natriuretic peptide (NT-proBNP) is an independent marker of renal anemia but is generally considered biologically inactive [308]. All data together suggest that atrial natriuretic peptide is a marker of negative outcomes, rather than a cause.

### 4.5. β_2_-Microglobulin

The interest in β_2_-microglobulin, the water-soluble extrinsic light chain of the Major Histocompatibility Class I (MHC I) molecules, was raised when it was demonstrated to be a major constituent of bone and joint amyloid deposits, which are at the origin of a devastating disorder of the locomotor system in renal patients, especially in those treated by dialysis [309]. Although the disease essentially has been linked to long-term renal replacement therapy, lesions were shown at the end of previous century to be present already from the second year of dialysis on, both in hemodialysis [310] and peritoneal dialysis patients [311]. Especially increased patient age at the onset of dialysis and the use of small pore complement activating cellulosic dialysis membranes as compared to large pore biocompatible AN69 membranes, seemed to affect the frequency of the disorder [312]. Interestingly, prevalence tended to decrease over the years following the detection of the problem [313]. This evolution was attributed to the introduction of large pore dialyzers and even more, to the improvement in quality of dialysis water [313].

A specific role in amyloid generation is attributed to the glycosylation of β_2_-microglobulin by e.g., imidazolone [314] and N^ε^-carboxymethyllysine [315]. Glycosylated β_2_-microglobulin, in contrast to the naïve molecule, had a pro-inflammatory impact on human monocytes, with the potential to lead to bone and joint destruction [316]. More recent studies assessed the mechanisms of deposition of amyloid fibrils and point to the generation of specific component species prone to aggregation [317] and conformational changes attributable to the binding of calcium [318]. β_2_-microglobulin fibrils then further affect function and/or viability of osteoblasts, osteoclasts and chondrocytes [319].

In various observational studies both in non-dialyzed [320] and dialyzed CKD patients [321,322,323,324] and even in populations not specifically selected because of CKD [325,326,327] β_2_-microglobulin has been associated to hard outcomes or to surrogate outcomes like parameters of inflammation [323], vascular stiffness [323,324] or bone remodeling [326,327]. Although in some of these studies adjustments were made for GFR [320,325], it is difficult to support unequivocally the causative role of β_2_-microglobulin from these data as the concentration of the molecule may sometimes have functioned as a surrogate for (residual) renal function.

From these observations, the question arises whether, apart from its role in renal amyloidosis, β_2_-microglobulin should be considered as a toxin by itself or just simply as a marker of renal failure. A number of studies point in the direction of a biological (toxic) effect. In a proteomic study searching for a biomarker of peripheral artery disease, plasma β_2_-microglobulin came out as a significant discriminator [328]. Kidney function was however lower in the affected patients, so that also in this study it remained unclear whether β_2_-microglobulin was a patho-physiologic player, or whether it merely acted as a marker of kidney function, resulting in an only indirect link to vascular disease. An in vitro study showed a link between β_2_-microglobulin and modifications in the molecular structure of the red blood cell plasma membrane, potentially affecting erythrocyte shape and their removal from the circulation [329]. In vivo and in vitro experimental studies showed an impact on osteoclast formation [330] and upregulation of TNF-α and Interleukin-6 (IL)-6 generation [330], epithelial-to-mesenchymal transition, be it in a cancer model [331] and a neurotoxic effect [332]. In a milestone study assessing functional neurologic changes during ageing, β_2_-microglobulin was identified as a pro-ageing factor, impairing hippocampal cognitive and regenerative function [333]. However, in a study assessing the immunologic impact of β_2_-microglobulin by using a commercially available β_2_-microglobulin batch, the originally found strong pro-inflammatory effect disappeared after purification of the β_2_-microglobulin solution by in vitro dialysis [334], suggesting a contaminant in the commercially available product. Hence, any finding of a biological effect of β_2_-microglobulin and by extension of any uremic toxin, should be regarded with care.

β_2_-microglobulin is considered as a prototypic middle molecule and is traditionally used as a marker of middle molecule removal, although both in pre-dialysis CKD [335] as in dialysis patients [336], β_2_-microglobulin behavior did not necessarily conform with that of other middle molecules. Removal in a hemodialysis setting can only be obtained by using large pore membranes (so-called high-flux dialyzers) [337,338]. Removal can be further increased by adding convection (hemodiafiltration) [339,340,341] but also by extending the length of dialysis [342]. A strong decline in pre-dialysis β_2_-microglobulin was obtained when several of these features were combined in extended daily hemodiafiltration [343]. 

Overall, the clinical suggestion of a link between β_2_-microglobulin and negative outcomes is strong but may be confounded by (residual) renal function and selection bias. Also in view of the not too extensive biochemical proof of negative action of the compound, some may remain not completely convinced regarding the toxicity of β_2_-microglobulin. 

### 4.6. β-Endorphin

β-Endorphin concentrations are increased in uremia [344]. In analogy to cholestatic colitis, uremic pruritus has been linked to stimulation of central µ-opioid receptors by accumulated endorphins, a hypothesis strengthened by the improvement of uremic itching in a randomized cross-over trial by the administration of the µ-opioid receptor blocker naltrexone [345]. This study was however never confirmed [346] and both pre- and post-dialysis plasma β-endorphin levels could not be correlated to clinical itching scores in dialysis patients [347]. For the rest, there is only limited data on the biological impact of β-endorphin. In rat adrenal gland cells, an in vitro study from 1981 showed that β -endorphin induced aldosterone generation [348]. 

### 4.7. β-Lipotropin

β-Lipotropin is the precursor of β-endorphin and is also retained in uremia [344]. Consequently very likely all above conclusions about β-endorphin can also be extended to β-lipotropin, including its effect on the adrenal gland [348].

### 4.8. Cholecystokinin

Cholecystokinin has been associated to disturbed appetite of uremia and thus malnutrition [349,350]. In PD patients, the molecule measured in fasting state was correlated to a feeling of fullness and a lack of hunger perception [351]. In contrast, another study in PD patients found no difference in cholecystokinin levels between anorexic and non-anorexic patients and cholecystokinin levels were positively related to surrogate markers of nutritional status, such as serum albumin or triglycerides [352].

### 4.9. Complement Factors D and Ba

Complement factor D as one of the elements of the complement cascade is retained in uremia [1,353] and has since long been considered to be one of the factors contributing to the pro-inflammatory status of uremia [1,2]. Retention is at least in part linked to a failure of renal catabolism [354]. In ESKD, extra-corporeal treatment over large pore membranes (hemofiltration) could remove a substantial fraction of complement factor D [355], which in part may be related to adsorption, as demonstrated for the polyacrylonitrile dialysis membrane [356]. Also, the immunosuppressive complement factor Ba is retained in uremia [357] and also this compound is removed by large pore extracorporeal strategies [355]. In view of this parallel retention and removal of a pro-inflammatory and an immunosuppressive factor, the final impact on the immunological balance of these complement factors remains unclear.

### 4.10. Cystatin C

Cystatin C is best known as marker of kidney function and cystatin C based estimated GFR values have been shown to better correlate to hard outcomes than creatinine-based formulations [358,359,360]. Although originally considered as being unconfounded by other conditions, later studies showed several demographic confounding factors [361,362]. Cystatin C is also produced by hematologic cell lines [255] and has been proposed as a biomarker of aggressive hematologic tumors [363]. To the best of our knowledge, there are almost no data pointing to a toxic effect of cystatin C in a uremic setting. It is a major regulator of protease activity, prevents tumor metastasis and is neuroprotective [255]. In the context of Alzheimer, it protects against amyloid β cytotoxicity [364] although it also seems to play an amyloidogenic role [255].

### 4.11. Cytokines

The cytokines are extensively reviewed in another article of this special issue [296]. That publication summarizes studies on the uremic toxicity of IL-1β, IL-6 and TNF-α with data up to the level of RCTs and on IL-18 and IL-8 with data up to the experimental and/or clinical observational level [296]. IL-10 is an anti-inflammatory cytokine and should not be considered as a uremic toxin [296]. However, because of this property it may be involved in the uremic propensity to infection. This review furthermore also contains information on the adipokines leptin, resistin and adiponectin that are discussed elsewhere in the present review.

### 4.12. Endothelin

Endothelins are strong vasoconstrictors [365]. Their concentration was shown to be increased in CKD [366]. Endothelin-1, via activation of one of its receptors, the endothelin-A (ETA) receptor, plays a deleterious role by increasing blood pressure and causing renal inflammation, oxidative stress, vascular shear stress, arterial stiffness, endothelial dysfunction and atherosclerosis [367,368]. Vascular endothelin production in CKD may be attenuated by antihypertensive treatment [369]. Renal production of endothelin-1 is however only reduced by angiotensin receptor blockade, a mechanism that may contribute to the renal protective effect of these drugs [369]. In experimental studies in CKD, specific blockade of the endothelin receptors reduced smooth muscle cell proliferation [370] and increased endothelial capillary density [371]. In a short term randomized cross-over study in CKD patients, Endothelin-A receptor blockade resulted in decreases of plasma urate, ADMA, proteinuria, hypertension and vascular stiffness [372,373]. Doubts on the long-term use of these agents was however raised when 3 to 6 months of treatment were not only associated with a reduction of proteinuria but also with a rise in mortality due to fluid retention [374] but maybe these problems could be overcome in future by using lower doses or more selective blockers [375]. Nevertheless, in spite of the contradictory results of hard outcome studies, enough proof-of-concept data have been provided to point to endothelin as a compound with major negative biological impact.

### 4.13. Fibroblast Growth Factor-23 (FGF-23)

The toxicity of FGF-23, a relative newcomer in the field of uremic toxicity research, is reviewed in depth in the current special issue [376]. Interestingly, this compound that in observational studies appeared to correlate extensively with negative outcomes, has now been shown to exert direct toxicity as well [376]. Hence, FGF-23 is not a mere marker of CKD and its hard outcomes but it can now also be considered as one of the potential culprits of the uremic syndrome. 

### 4.14. Ghrelin

Ghrelin levels have definitely been shown to be increased in CKD [377,378,379,380]. Ghrelin is known as an orexigen playing a role in meal initiation [381] and as such seems to oppose the trend for malnutrition of most CKD patients. However, the molecule has also been linked to several other negative biological activities, such as restricted insulin release [382] and blunted insulin sensitivity [383], increases in triglyceride content in the liver [384], mitochondrial oxidative enzyme activities [384], anxiogenic response to stress [385], increase in serum corticosterone levels [385], release of growth hormone [386] and increases in sodium reabsorption [387,388]. On the other hand, ghrelin also has been linked to a rise in cardiac index and stroke volume index [389]. 

Clinically, low ghrelin levels have been associated with inflammation and cardiovascular risk in hemodialysis patients [390]. On the other hand, ghrelin correlated negatively with body mass index [377]. In PD patients weight gain over time was associated to a decrease of ghrelin levels [377]. In another study, ghrelin levels were inversely correlated to nutritional makers suggesting increases in ghrelin at low dietary intake [380]. The acute response to oral feeding in CKD was however markedly blunted versus controls [379]. A possible explanation for the seeming paradox between uremic malnutrition and elevated ghrelin can be found in the finding that ghrelin is present in the body in two forms, the orexigen acylated ghrelin and desacylated ghrelin which induces a negative energy balance by decreasing food intake and gastric emptying [391]. The balance between these two forms has repeatedly been shown to be disrupted in CKD, with more prominent retention of desacyl ghrelin [378,392,393]. Plasma desacyl ghrelin was significantly higher in anorexic hemodialysis patients [392]. 

### 4.15. Glomerulopressin

The only functional effect of glomerulopressin that could be found was an increase of glomerular filtration rate and potassium excretion [394].

### 4.16. Immunoglobulin Light Chains

Although typical for multiple myeloma and other hematologic malignancies [395], the concentration of the immunoglobulin light chains is also above normal in patients with CKD not suffering from those conditions [396,397]. These molecules have been depicted as markers to inflammation but possibly also as pathogenetic factors [398,399]. Experimental studies applying immunoglobulin light chains at concentrations as observed in CKD demonstrated that they decreased polymorphonuclear glucose uptake and chemotaxis [400] but also leukocyte apoptosis, a factor possibly contributing to chronic inflammation [401]. After their filtration by the glomeruli, immunoglobulin light chains are incorporated via the cubilin receptors into the renal proximal tubular cells [402] where they are catabolized [403] and where they tend to accumulate if the system is overloaded [404] to cause morphologic changes [405] and kidney injury [406], be it essentially at the extremely high concentrations of hematologic malignancies [407]. Their toxicity may be further enhanced by glycosylation [408]. In observational studies in CKD, their concentration has been associated to inflammation and vascular calcification in one study [409] and mortality in another one [396], although in the first study adjustment for well-known cardio-vascular risk factors abolished the link to mortality [409]. In ESKD, immunoglobulin light chains can only be removed by dialysis via large pore high-flux membranes, although treatment does not reduce levels to normal [397,410]. Substantially better removal could be obtained by using extremely open high cut-off (HCO) dialyzer membranes but at the expense of considerable albumin losses [411]. The novel medium cut-off (MCO) membranes are an intermediate between classical high-flux and HCO, allowing better albumin conservation. For removal of immunoglobulin light chains, they are more efficient in a hemodialysis mode than classical high-flux membranes in hemodiafiltration [412].

### 4.17. Modified Lipids and Lipoproteins

Oxidation does not only modify proteins but also lipids and next to oxidation, there are also other post-translational lipid modifications at play in CKD, such as glycation and carbamylation. As summarized by a review in this special issue, they play a role in inflammation, apoptosis, endothelial dysfunction and accelerated atherosclerosis [413].

### 4.18. Leptin

Leptin is a satiety hormone that is part of the review on the cytokines in this special issue [296]. In spite of ample experimental arguments in favor of its biological activity [414], clinical data are contradictory and not convincing, leading to the conclusion in the above mentioned publication that its status as a uremic toxin is debatable [296]. 

### 4.19. Macrophage Colony Stimulating Factor

Macrophage Colony Stimulating Factor (or Colony Stimulating Factor-1—CSF-1) was originally described as a promotor of proliferation and differentiation of hematologic progenitor cells [415] but soon appeared to stimulate also the activity of mature leukocytes, by stimulating neutrophil metabolism and cytotoxicity [416,417] as well as monocyte cytokine production [418]. Hence, Macrophage CSF might be a key factor in inflammatory conditions [419], including CKD [420]. However, also beneficial effects have been attributed to it, such as regression of atherosclerotic lesions [421], recovery from leukocytopenia [422] and kidney repair from ischemia [423]. After addition of this component as an adjuvant to a hepatitis B vaccine, an improved response to vaccination was observed in hemodialysis patients [424].

### 4.20. Methionine-Enkephalin

Methionine-enkephalin is another opioid of which the concentration is increased in CKD [425,426]. Methionin-enkephalin plays a role in suppressing the feeling of pain [427,428], enhances the secretion of several hormones [429], suppresses renal nerve activity [430] and inhibits jejunal fluid secretion [431]. 

### 4.21. Neuropeptide Y

Neuropeptide Y, a known major orexigen [349,350,432], is retained in CKD [433,434]. In peritoneal dialysis patients, low neuropeptide Y levels are associated with anorexia [352]. In the nervous system, neuropeptide Y inhibits the release of the neurotransmitter acetylcholine in nodose neurons [435]. Neuropeptide Y also acts as a renal vasoconstrictor by suppression of adenylate cyclase activity, a regulator of a wide array of cellular processes in vascular smooth muscle cells [436]. Likewise, neuropeptide Y also inhibits cardiac adenylate cyclase [437] and the combination of high leptin, peptide YY and neuropeptide Y together with low ghrelin has in the hemodialysis population been linked to hypertension, inadequate vasodilation and cardiac hypertrophy and hence could predispose to cardiovascular events [438]. In an observational study, neuropeptide Y predicted cardiovascular complications in ESKD [439]. 

### 4.22. Orexin A

Orexin A is another orexigen [440] of which the messenger RNA expression is suppressed in CKD [441]. It was difficult to find data on its concentration in CKD and also the data on the biological impact are scarce, especially in uremia. Studies with normal kidney function and usually after central injection show a stimulation of testosterone production [442] and a rise in blood pressure and tachycardia in stressful conditions [443].

### 4.23. Parathyroid Hormone (PTH)

Disturbances in concentration of PTH, phosphate and FGF-23 are strongly linked to each other whereby changes in one factor impact generation of the other, often following an adaptive response mechanism [444,445]. The biological impact of PTH has extensively been studied and summarized and covers multiple aspects such as peripheral neuropathy, carbohydrate metabolism, hypertension, mitochondrial respiration and cardiac hypertrophy [445]. Yet, due to inherent PTH-resistance in CKD, PTH levels need to be more elevated than with normal kidney function before generating an effect [445]. In observational studies, both low and high PTH levels have been linked to increased mortality, with a more pronounced relationship for the low levels [445,446], which are linked to adynamic bone disease. Hence, the validity of parathyroid hormone as a marker of bone turnover and generator of cardiovascular disease has been questioned [447]. In two RCTs, parathyroid hormone lowering treatment with the calcimimetic Cinacalcet did not impact the primary endpoints of the studies [448,449] but influenced a number of surrogate endpoints such as vascular and cardiac valve calcification [448]. In a secondary analysis of one of these two trials, the EValuation Of Cinacalcet Hydrochloride (HCl) Therapy to Lower CardioVascular Events (EVOLVE) study, Cinacalcet appeared to lower FGF-23 and these reductions were linked to a lower number of cardiovascular events and death [450]. As the rise in FGF-23 precedes the rise in parathyroid hormone and serum phosphate, the question may be raised whether the rise in FGF-23 is not the primary pathophysiologic event in the development of secondary hyperparathyroidism [451]. However, FGF-23 neutralization with antibody in mice with kidney failure improved hyperparathyroidism but increased mortality [452]. All data taken together, the pathophysiologic role of parathyroid hormone in CKD cannot be supported unequivocally.

### 4.24. Pentraxin-3

Pentraxin-3 is an acute phase protein produced by a variety of cells in response to inflammatory signals [453]. The pentraxins are considered to be useful biomarkers of systemic inflammation [454]. Pentraxin-3 levels have repeatedly been shown to be increased in CKD [455,456,457]. Direct proof of a biological effect is scarce. One study linked Pentraxin-3 to epithelial-to mesenchymal transition and renal fibrosis [458]. In addition, pentraxin-3 was found in foam cells of advanced atherosclerotic lesions and in leukocytes infiltrating atherosclerotic plaque [459]. However, most studies linking Pentraxin-3 to negative outcomes are clinical observational and associate the molecule to endothelial dysfunction [457,460], cardiovascular disease [455,456,457,460,461], protein energy wasting [455,457] and in a study focusing on the elderly, also incident CKD [462]. In unselected hypertensive diabetics, antihypertensive treatment improved flow-mediated vessel dilation and normalized proteinuria but normalized Pentraxin-3 as well [463,464]. Moreover, in hypertensive type 2 diabetics, the improvement in flow-mediated vasodilation related to antihypertensive treatment was independently associated with Pentraxin-3 concentration [464]. Yet, in animal experiments in mice, Pentraxin-3 infusion attenuated renal damage in diabetic nephropathy [465], whereas knock-out mice lacking pentraxin-3 showed more vascular inflammation and macrophage accumulation within atherosclerotic plaque [466]. In obese CKD patients pentraxin-3 levels were low in spite of enhancement of other inflammatory markers [467]. In a functional ex vivo study, subcutaneous fat tissue pentraxin-3 mRNA was inversely correlated to ADMA concentration and basal resistance artery tone [468]. The latter studies suggest that in some circumstances, pentraxin-3 may play a counter regulatory role in endothelial damage. On the other hand, in the observational arm of the same study, adipose pentraxin-3 mRNA was increased in CKD patients with cardiovascular disease but the association disappeared after adjustment [468]. Overall, there seem to be as much arguments in favor as in disfavor of the biological toxicity of pentraxin-3. 

### 4.25. Peptide YY

Peptide YY is an anorexigen [469,470] and inhibitor of gastric motility [471]. Elevated levels have been recorded in CKD patients on peritoneal dialysis [472] and on hemodialysis [438], although its postprandial secretion was markedly decreased as compared to healthy controls [472]. Studies on biological effects are scanty. Infusion of peptide YY was shown to decrease glomerular filtration rate, plasma renin activity and aldosterone levels, whereas urinary sodium excretion was raised [473]. It has been hypothesized that the combination of high leptin, peptide YY and neuropeptide Y together with decreased ghrelin would predispose to hypertension, impaired vasodilation and cardiac hypertrophy [438], although the decrease of renin and aldosterone mentioned above [473] may cast some doubts on the contribution of peptide YY into this. On the other hand, on the pancreatic level, peptide YY seems to be essential for beta cell survival, insulin secretion and glycemic control [474,475].

### 4.26. Prolactin

Hyperprolactinemia is common in CKD and has been observed since many years [476,477,478,479]. As a hormone involved in reproductive behavior [480] it could play a role in sexual dysfunction of uremia [481] but treatment with bromocryptin could suppress prolactin but did not correct sexual dysfunction in hemodialysis patients of both genders [479]. There are only limited data on the biological action of prolactin, which has been linked to platelet activation [482]. On the other hand, under physiologic non-uremic conditions, prolactin has a homeostatic role, e.g., in maintenance of immune function, osmoregulation, suppression of angiogenesis and by extension possibly also in tumor genesis [480]. One observational clinical study linked prolactin to markers of endothelial dysfunction and cardiovascular morbidity and mortality [476]. A similar relation with major cardiovascular events has also been observed in males without CKD but with erectile dysfunction [483].

### 4.27. Resistin

Resistin is included in the review on cytokines which is part of this special issue [296] but is not considered a uremic toxin. In spite of a few suggestive experimental studies, no link with hard outcomes in CKD could as yet be demonstrated [296].

### 4.28. Retinol Binding Protein

Retinol binding proteins are not only transporters of retinoids but also function as regulators of retinoid metabolism and disposition [484]. They have extensively been studied in CKD and were repeatedly shown to be elevated [485,486,487,488,489], although levels do not correlate as well with GFR than Cystatin C or β_2_-microglobulin [335,490]. Contradictory biological functions have been attributed to this molecule. On one hand, retinol binding protein has been shown to induce apoptosis and inflammatory reaction in human endothelial cells [491,492]. On the other hand, retinol binding protein isolated from acute kidney failure patients inhibited polymorphonuclear cell function and apoptosis [401]. Quite a number of observational studies in specific populations not necessarily suffering from CKD link retinol binding protein to cardiovascular disease [493], carotid intima thickness [494], the metabolic syndrome [495,496,497] and obesity [496]. Only a few observational studies assessed links of retinol binding protein with outcomes in specific populations suffering from CKD. In one study serum levels were associated to components of the metabolic syndrome [488]. In a population of type 2 diabetics with and without CKD, serum retinol binding protein was however not related to carotid intima thickness [498]. On the other hand urinary retinol binding protein was inversely related to kidney function and cardiovascular risk factors [499] and was also increased in proportion with the degree of renal radiation damage in mice [500]. 

### 4.29. Visfatin

Visfatin is included in the review on cytokines which is part of this special issue [296]. Also, this molecule was discarded as a potential uremic toxin, because of contradictory clinical data [296].

### 4.30. Summary

Like with the two other large groups of uremic retention solutes, these data show that also the middle molecules have the capacity to affect a number of biological systems that contribute to the uremic syndrome. The average number of experimentally affected systems (2.34 ± 1.57) is significantly lower (P = 0.012) than for the protein bound compounds but comparable to the small water-soluble compounds. The systems affected by the largest number of toxins are cardiovascular system (n = 21), inflammation (n = 16) and fibrosis (n = 12) (Figure 2E, Supplementary Materials Table S3). Of note, protein energy wasting, without directly involved molecules within the two other groups, appeared to be affected by 4 of the middle molecules, pointing to the many peptidic structures that function as an anorexigen. There are relatively few middle molecules that have been demonstrated to affect more than 4 biological systems (Figure 2F, Supplementary Materials Table S3), suggesting a relative scarcity of experimental studies in this group of molecules. The molecules with the largest impact are: β_2_-microglobulin (6 affected systems) and ghrelin, leptin and parathyroid hormone (all n = 5). 

The average evidence score (Table 6) is 2.06 ± 1.19 (maximum possible = 4). For this characteristic, the list is headed by β_2_-microglobulin, interleukin-6, TNF-α and FGF-23 (all n = 4). 

## 5. Discussion

This review summarizes the current knowledge about the biological impact (toxicity) of uremic retention compounds. From this analysis, it appears that there is a plethora of compounds that exert some degree of toxicity. In total 8 groups of compounds and 63 individual compounds (20 small water-soluble, 16 protein-bound and 35 middle molecules) are reviewed of which 66 (93%) are modifying the activity of at least one functional system. Hence, the uremic syndrome is, with regards to the patho-physiologic impact of the retained solutes, a multifactorial condition. It seems highly improbable that one day a “golden bullet” would be identified, of which the removal would solve the entire problem. We are rather confronted with groups of compounds depending on various characteristics (protein bound vs. not; high molecular weight vs. low; hydrophilic vs. hydrophobic; intestinally generated vs. not; secreted by the renal tubules vs. not) that should be handled together if it comes to decrease their concentration. 

When we consider the number of functional systems disturbed per molecule, the highest score of 7 on 11 systems considered is reached by one group of small water-soluble compounds (the polyamines), one group of protein bound solutes (the AGEs) and one individual protein bound compound (p-cresyl sulfate) and no middle molecule. The second highest score of 6 is reached by one small water-soluble compound (uric acid), one protein bound compound (indoxyl sulfate) and one middle molecule (β_2_-microglobulin). Obviously, it is easier to reach a high score for solutes that are considered as a group (e.g., the AGEs and the polyamines) than for the other solutes that are considered individually. In addition, the score per solute depends on the frequency a solute or group of solutes was studied and per physico-chemical type, on the number of solutes or groups of solutes included in that category. Taking these into account, it could be concluded that the number of high scores is limited for the middle molecules in spite of the fact that it actually happens to be the largest group of individual entries (n = 35). This impression is confirmed by the, be it not always significant lower average score for the number of affected systems (2.34 ± 1.57 vs. 2.65 ± 1.95 and 3.69 ± 1.96 for the small water-soluble compounds and the protein bound compounds, respectively—Table 7). This points to the relatively low number of experimental studies on the middle molecules in general, either due to a low intrinsic number of investigations, maybe since their toxicity is automatically taken for granted, or to a reporting bias with negative results remaining unpublished. On the other hand, the superiority of protein bound solutes with regards to experimental data seems largely lost when the overall evidence score is taken into account (Table 7). 

Thus, our data suggests that the number of experiments assessing the biochemical impact of individual retention compounds might be increased, especially for the middle molecules and to a certain extent also for the small water-soluble compounds. Indeed, whereas the protein bound solutes have largely been neglected until the mid-nineties of previous century, their analysis has received a substantial boost since then. In addition, however, we also stress the importance, for researchers, to report negative results and for journals, to publish them.

In this context, it should also be noted that, whereas most uremic toxin research has up to now focused on single toxins, in real life they are present together in the body. The problem of mutual interaction of toxins can only be solved by studying solute panels, although this approach may be hampered by problems of solubility if several molecules are combined together and correct uremic concentrations have to be obtained [501].

For a total number of 71 solutes or solute groups, a negative effect per functional system was reported for 41 molecules or groups on the cardiovascular system (58% of considered solutes), followed by 37, 26, 23 and 15 molecules (52%, 37%, 32% and 21%) for inflammation, metabolic function, fibrosis and neurotoxicity respectively. All these findings point to a multifactorial disintegration of some of the key elements which define morbidity and mortality of our patients on dialysis and already a long time before dialysis is initiated (cardiovascular damage, inflammation and fibrosis as a cause of heart failure and progression of CKD). However, because of this patho-physiologic importance, these systems may also have been assessed more frequently, hence leading to an underrepresentation of the other systems, although they are important as well for the quality of life of CKD patients. In general, it seems that we could retrieve for this review only few studies that assessed quality of life or factors defining quality of life in uremia (e.g., pruritus, insomnia). This lack of patient-centered analyses is another shortcoming that could be corrected in future.

A direct effect on protein energy wasting (PEW) was conveyed only 4 times but some of the other frequently reported systems (inflammation, cardiovascular disease, neurotoxicity, infection) as such are an indirect cause of protein energy wasting.

For the overall evidence score (experimental + clinical, with correction for results indicating no effect or a beneficial effect) the maximum score of 4 was reached by 3 small water-soluble compounds or groups of compounds (ADMA, SDMA and the carbamylated products) and 4 middle molecules (β_2_-microglobulin, TNF-α, interleukin-6 and FGF-23) but only in two protein bound solutes or solute groups (p-cresyl sulfate and the kynurenines) (Table 8). In part, the latter finding could be linked to the number of protein bound solutes considered which is lower than that of the two other groups. Yet, this figure is markedly low as compared to the experimental data that were retrieved. Hence, our data point to a relative shortage of clinical hard outcome studies on some of the protein bound uremic toxins (e.g., indole acetic acid, phenyl acetic acid, p-OH hippuric acid). On the other hand, the score was also often downgraded because of too many observational studies showing no association with hard outcomes. Such neutral results might be real but may also be the consequence of insufficiently powered studies or confounding by unrecognized or unknown factors. This is the reason why in Table 8 summarizing the highest scoring solutes we also included solutes with score 3.

When listing the highest ranking molecules with regards to their toxicity (overall evidence score ≥3, experimental score ≥5) the following molecules or groups of molecules could be retained (Table 8): for the small water-soluble compounds, ADMA, uric acid and TMAO; for the protein bound compounds, p-cresyl sulfate, kynurenines, AGEs, indoxyl sulfate, indole acetic acid, phenyl acetic acid; for the middle molecules, β_2_-microglobulin, ghrelin, parathyroid hormone.

RCTs are currently accepted to offer the highest level of evidence for a given impact. Although there are some RCTs on interventions to decrease the concentration of specific toxins, they are few, largely due to the low number of available interventional therapies that specifically can change concentration of a given uremic toxin. In view of the multifactorial pathophysiology that can be understood from this and other reviews [3], one may wonder whether it makes much sense to further consider specific interventional trials to decrease one single solute in the context of uremia, unless to offer proof of concept of toxic effects. In addition, such therapies are not always innocent. Allopurinol e.g., may reduce uric acid but any positive effect that is generated might be partially offset by its complications, such as Stevens-Johnson syndrome or leukocytopenia. Hence, it might be more useful to try to influence the concentration or metabolic impact of groups of molecules with similar characteristics. Thus, we still remain in need of randomized controlled trials in the area of CKD, or alternatively, of observational trials with optimized corrective strategies for residual confounding but they should preferentially be focused on larger groups of molecules. Observational studies might have an advantage on controlled studies by better mimicking real life conditions, in an area where many randomized controlled trials turned out negative at primary analysis for hard outcomes [161,162,287,288,289,338,449,502,503]. 

This is the first analysis of the biological capacity of the majority of currently known uremic compounds since almost two decades. Not only data from the nephrologic literature but also a large array of non-nephrologic journals were taken into account. In addition, when neutral or beneficial results had been reported, these were also taken into consideration in our scoring systems.

This analysis, although comprehensive, also has a number of drawbacks. First, we may have failed to include some compounds. However, as we started from existing uremic databases [1,5] the risk that this would have been the case is relatively low and if it happened, it is unlikely that one of the currently known major contributors to the uremic syndrome will have been missed. Second, we may have overlooked a number of studies showing a biological impact, since this analysis is not based on a systematic review. Systematic reviews are strenuous endeavors, simultaneously involving several researchers for substantial time periods, so that for practical reasons they are to be restricted to a limited number of toxins [20] and should be considered as an inappropriate tool for a comprehensive review such as the present one. In addition, if some references are missing, it is more likely to have occurred for those solutes that have been studied extensively, than for those with a limited number of data. For that reason, it is improbable that substantial shifts would occur in the scores of the individual solutes and even more in the global assessment per large group of solutes if the literature base for their analysis would have even been larger than it already is with close to 500 publications included in the reference list. However, in future, it might be useful to consider more in depth systematic analysis of solutes scoring high in this assessment, to see whether their present score was justified. Likewise, we did not check whether in all cited studies the correct concentrations as observed in uremia were applied, which would be another element to be assessed by a systematic review. However, we are convinced that this review and especially the classification offered in Table 8, will be helpful in future to select the most important toxins if such systematic reviews were planned. Finally, the perception emanating from this review may be confounded by publication bias. On one hand, negative results may have retained insufficient attention, on the other hand the impression of the toxicity of some other solutes may seem overwhelming, because they were more frequently the subject of studies.

## 6. Conclusions

We thus believe that this review will offer a useful reference work giving a better insight in the complex picture of uremia to those interested and at the same time hope it is a stimulus to fill in the gaps in knowledge that still exist and that it could serve as a good reference work for future uremic toxicity analyses and for selecting mechanisms for future drug development in the treatment of the uremic syndrome. Our ranking system may also be of help for defining the target molecules in removal studies and for developing novel methods improving this removal or removing molecules that up till now received insufficient attention.

The scores are also likely to stimulate clinical and/or translational research on specific uremic toxins or groups of toxins by clarifying for which compounds and for which physiologic systems data are abundant and for which not.

We demonstrated a kaleidoscopic picture of the many compounds involved in defining the pathophysiologic process that ultimately results in the uremic syndrome. We summarize the biological/biochemical and clinical impact of 71 uremic retention solutes that have been assessed over the past half of a century. A complex picture emerges whereby almost all considered solutes affect one or more functions that contribute to the uremic syndrome and its complications, leading to the increased morbidity and mortality of CKD. Of the affected mechanisms, especially the susceptibility to cardiovascular damage, inflammation and fibrosis appeared to be targeted but these systems have presumably also been studied most extensively. Some discrepancies between experimental and clinical data became apparent, leading sometimes to a downgrade of positive looking experimental scores. In addition, it became clear that the quantity of experimental data for the middle molecules and to a certain extent also for the small water-soluble compounds could be larger, whereas clinical data are missing for several of the protein bound solutes in spite of quite robust experimental data being available. In view of the apparently multifactorial nature of uremia, it seems illusory to think that decreasing the concentration of one single solute will improve outcomes and probably attention should concentrate on decreasing the concentration of groups of solutes. More attention could be paid to quality of life aspects.

## Figures and Tables

**Table 1:** The different classes of uremic toxins as used in this publication.

Class of Molecules	MW Range	Prototype	MW Prototype
Small water-soluble compounds	<500 Da	Urea	60
Protein bound compounds	Mostly < 500 Da	Indoxyl sulfate	213.2
Middle molecules	≥500 Da	β_2_-microglobulin	11.818

MW: molecular weight; Da: Dalton.

**Table 2:** List of toxins considered in this publication per class.

Small Water-Soluble Compounds	Protein Bound Compounds	Middle Molecules
Guanidine compounds	AGEs	Adrenomedullin
-Guanidinosuccinic acid	AOPPs	Adiponectin
-Methylguanidine	CMPF	Angiogenin
-Guanidine	Cresols	Atrial natriuretic peptide
-Creatine	-P-cresyl sulfate	β_2_-microglobulin
-Guanidino acetic acid	-P-cresyl glucuronide	β-endorphin
-γ-Guanidino butyric acid	Hippurates	β-lipotropin
-ADMA	-Hippuric acid	Cholecystokinin
-SDMA	-P-hydroxy hippuric acid	Complement factor D
Oxalate	-O-hydroxy hippuric acid	Complement factor Ba
Phenylacetylglutamate	Homocysteine	Cystatin C
Methylamines	Indoles	Interleukin-1β
-(Mono)methylamine	-Indoxyl sulfate	Interleukin-18
-Dimethylamine	-Indoxyl glucuronide	Interleukin-6
-Trimethylamine	-Kynurenine	Tumor Necrosis Factor-α
-Trimethylamine-N-Oxide	-Kynurenic acid	Interleukin-8
Sulfuric compounds	Phenols	Interleukin-10
-Lanthionine	-Phenyl sulfate	Endothelin
Myoinositol	-Phenyl acetic acid	FGF-23
2PY	Quinolinic acid	Ghrelin
Polyamines		Glomerulopressin
-Acrolein		Immunoglobulin light chains
-Putrescine		Lipids and lipoproteins
-Spermine		Leptin
-Spermidine		MCSF
Urea		Methionine-enkephalin
Carbamylated compounds		Neuropeptide Y
Cyanate		Orexin A
Ammonia		Parathyroid hormone
Uric acid		Pentraxin-3
Xanthine		Peptide YY
Hypoxanthine		Prolactin
		Resistin
		Retinol Binding Protein
		Visfatin

ADMA: Asymmetric Dimethylarginine; SDMA: Symmetric Dimethylarginine; 2PY: N-Methyl-2-Pyridone-carboxamide; AGEs: Advanced Glycation End Products; AOPPs: Advanced Oxidation Protein Products; CMPF: 3-Carboxy-4-Methyl-5-Propyl-2- Furanpropionic Acid; FGF-23: Fibroblast Growth Factor 23; MCSF: Macrophage Colony Stimulating Factor.

**Table 3:** System applied for scoring evidence.

Characteristic	Points
1–2 experimental studies showing toxicity	1
>2 experimental studies showing toxicity	2
≥25% of retrieved experimental studies showing no toxicity or benefit	−1
1–2 clinical studies* showing association with hard outcomes **	1
>2 clinical studies* showing association with hard outcomes **	2
≥25% of retrieved clinical studies* showing no association with hard outcomes ** or benefit	−1

* Observational studies and Randomized Controlled Trials (RCTs); ** Hard outcomes: mortality, cardio-vascular events, progression of CKD.

**Table 4:** Summary of evidence data available on the small water-soluble compounds.

Type of Evidence Points	Exp. Toxic. 1-2	Exp. Neutral or Benefit -1	Clin. Toxic. 1-2	Clin. Neutral or Benefit -1	Sum 4 (max)
Guanidines *	2	0	0	0	2
ADMA	2	0	2	0	4
SDMA	2	0	2	0	4
Oxalate	2	0	0	0	2
PAG	0	-1	2	0	1
MMA	1	0	0	-1	0
DMA	1	0	0	-1	0
TMA	2	0	0	-1	1
TMAO	2	0	2	-1	3
Lanthionine	1	0	0	0	1
Myoinositol	1	-1	0	0	0
2PY	1	0	0	0	1
Polyamines **	2	0	0	0	2
Urea	2	0	0	0	2
Carbamylated compounds **	2	0	2	0	4
Cyanate	2	0	0	0	2
Ammonia	1	0	0	0	1
Uric acid	2	0	2	-1	3
Xanthine	1	0	0	0	1
Hypoxanthine	1	0	0	0	1

Exp.: experimental; clin.: clinical (observational + Randomized Controlled Trials—RCTs); Toxic.: Toxicity; ADMA: Asymmetric Dimethylarginine; SDMA: Symmetric Dimethylarginine; PAG: Phenylacetylglutamine; MMA: Monomethylamine; DMA: Dimethylamine; TMA: Trimethylamine; TMAO: Trimethylamine-N-Oxide; 2PY: N-Methyl-2-Pyridone-carboxamide; * guanidines treated as one group, with the exception of ADMA and SDMA; ** polyamines and carbamylated compounds considered as one group.

**Table 5:** Summary of evidence data available on the protein bound compounds.

Type of Evidence Points	Exp. Toxic 1-2	Exp. Neutral or Benefit -1	Clin. Toxic 1-2	Clin. Neutral or Benefit -1	Sum 4 (Max)
AGEs *	2	0	2	-1	3
AOPPs *	2	0	1	-1 **	2
CMPF	2	0	1	-1	2
p-Cresyl sulfate	2	0	2	0	4
p-Cresyl glucuronide	1	-1	1	0	1
Hippuric acid	2	0	0	0	2
p-OH hippurate	2	0	0	0	2
o-OH hippurate	2	0	0	0	2
Homocysteine	2	0	1	-1	2
Indoxyl sulfate	2	0	2	-1	3
Indole acetic acid	2	0	1	0	3
Indoxyl glucuronide	1	0	0	0	1
Kynurenines *	2	0	2	0	4
Phenyl sulfate	1	0	0	0	1
Phenyl acetic acid	2	0	1	0	3
Quinolinic acid	2	0	0	0	2

Exp.: experimental; clin.: clinical (observational + Randomized Controlled Trials—RCTs); AGEs: Advanced Glycation End Products, AOPPs: Advanced Oxidation Protein Products; CMPF: Carboxy Methyl Propyl Furanpropionic Acid; p-OH hippurate: p-hydroxyhippurate; o-OH hippurate: o-hydroxyhippurate; * AGEs, AOPPs and kynurenines considered as one group; ** skewed by confounders due to measurement errors (see text).

**Table 6:** Summary of evidence data available on the middle molecules.

Type of Evidence Points	Exp. Toxic 1-2	Exp. Neutral or Benefit -1	Clin. Toxic 1-2	Clin. Neutral or Benefit -1	Sum 4 (max)
Adrenomedullin	1	-1	0	0	0
Adiponectin	0	0	2	-1	1
Angiogenin	2	0	0	0	2
ANP	1	-1	1	0	1
β_2_-microglobulin	2	0	2	0	4
β-endorphin	1	-1	1	0	1
β-lipotropin	1	-1	1	0	1
Cholecystokinin	0	0	2	-1	1
Complement factor D	2	0	0	0	2
Complement factor Ba	2	0	0	0	2
Cystatin C	1	-1	2	0	2
Interleukin-1β	2	0	0	0	2
Interleukin-18	2	0	1	0	3
Interleukin-6	2	0	2	0	4
TNF-α	2	0	2	0	4
Interleukin-8	2	0	1	0	3
Interleukin-10	2	0	1	-1 *	2
Endothelin	2	0	2	-1	3
FGF-23	2	0	2	0	4
Ghrelin	2	0	2	-1	3
Glomerulopressin	0	0	0	0	0
IgLC **	2	0	2	-1	3
Lipids & lipoproteins **^,^***	2	0	1	0	3
Leptin	2	0	0	0	2
MCSF	2	-1	1	0	2
Methionine-enkephalin	2	-1	0	0	1
Neuropeptide Y	2	-1	2	0	3
Orexin A	1	-1	0	0	0
Parathyroid hormone	2	0	2	-1	3
Pentraxin-3	2	-1	2	-1	2
Peptide YY	1	-1	0	0	0
Prolactin	1	-1	2	0	2
Resistin	2	0	0	0	2
Retinol Binding Protein	2	0	2	-1	3
Visfatin	0	0	2	-1	1

Exp.: experimental; clin.: clinical (observational + Randomized Controlled Trials—RCTs); ANP: atrial natriuretic peptide, TNF-α: Tumor Necrosis Factor-α, IgLC: Immunoglobulin Light Chains; MCSF: Macrophage Colony Stimulating Factor; * paradoxically correlated to consequences of inflammation whereas the biological action is anti-inflammatory; ** immunoglobulin light chains and lipids and lipoproteins considered as one group; *** modified—posttranslational.

**Table 7:** Mean scores for each major toxin group for the number of affected systems in experimental studies and for the average evidence score (experimental + clinical).

	Number of Affected Systems in Experimental Studies	Average Evidence Score
Small water-soluble compounds	2.65 ± 1.85	1.75 ± 1.29
Protein bound solutes	3.69 ± 1.96*	2.31 ± 0.95
Middle molecules	2.34 ± 1.57	2.06 ± 1.19

Original data on which the calculations are based are shown in Figure 2, Table 4, Table 5 and Table 6 and Supplemental Tables S1–S3. * *p* = 0.012 vs. middle molecules.

**Table 8:** Uremic toxins with the highest toxicity score.

Evidence Score: 4	Exp. Score	Evidence Score: 3	Exp. Score
p-Cresyl sulfate	7	AGEs	7
β_2_-Microglobulin	6	Indoxyl sulfate	6
ADMA	5	Uric acid	6
Kynurenines	5	Ghrelin	5
Carbamylated compounds	3	Indole acetic acid	5
FGF-23	3	Parathyroid hormone	5
Interleukin-6	3	Phenyl acetic acid	5
TNF-α	3	TMAO	5
SDMA	2	Retinol binding protein	4
		Endothelin	3
		IgLC	3
		Interleukin-1β	3
		Interleukin-8	3
		Neuropeptide Y	3
		Lipids & lipoproteins	2

Exp.: experimental; ADMA: Asymmetric Dimethylarginine; FGF23: Fibroblast Growth Factor-23; TNF-α: Tumor Necrosis Factor; SDMA: Symmetric Dimethylarginine; AGEs: Advanced Glycation End Products; TMAO: Trimethylamine-N-Oxide; IgLC: Immunoglobulin Light Chains.

## Supplementary Materials

The following are available online at www.mdpi.com/2072-6651/10/1/33/s1, Table S1: Experimental effects observed for the small water-soluble compounds, Table S2: Experimental effects observed for the protein bound compounds, Table S3: Experimental effects observed for the middle molecules.
