# Supplementary material for: Biochemical and Clinical Impact of Organic Uremic Retention Solutes: A Comprehensive Update
Source: Toxins (Basel). 2018 Jan 8;10(1):33. doi: 10.3390/toxins10010033 (PMC5793120; doi:10.3390/toxins10010033)
Supplement: Supplementary file 1 [file toxins-10-00033-s001.pdf]

Supplemental table 1: Experimental effects observed for the small water soluble compounds

	Inflammation	*CVD*	*CKD-MBD*	*Fibrosis*	*Infection*	*Insulin resistance*	*Metabolic function*	*PEW*	*Neurotoxicity*	*Thrombogenicity*	*Hematology*	*Sum*
**Guanidines***	■	■	■		■				■			5
ADMA	■	■							■		■	4
SDMA	■	■										2
Oxalate	■	■					■					3
PAG												0
MMA		■										1
DMA							■					1
TMA							■		■			2
TMAO	■	■		■			■			■		5
Lanthionine							■					1
Myoinositol									■			1
2PY							■					1
**Polyamines****	■	■			■	■	■		■		■	7
Urea	■	■				■						3
**Carbamylated compounds****	■	■		■								3
Cyanate		■				■				■		3
Ammonia	■											1
Uric acid	■	■		■	■	■	■					6
Xanthine			■		■							2
Hypoxanthine			■		■							2
Sum	10	11	3	3	5	4	8	0	5	2	2	

Metabolic function includes tubular function; the term fibrosis also covers progression of CKD; CVD: Cardiovascular Disease; CKD-MBD: chronic kidney disease – metabolic bone disease; PEW: protein energy wasting; ADMA: Asymmetric Dimethylarginine; SDMA: Symmetric Dimethylarginine; PAG: Phenylacetylglutamate; MMA: Monomethylamine; DMA: Dimethylamine; TMA: Trimethylamine; TMAO: Trimethylamine-N-Oxide; 2PY: N-Methyl-2-Pyridone-carboxamide; *: guanidines considered as one group with the exception of ADMA and SDMA; **: polyamines and carbamylated compounds considered as one group. Colored for demonstrated effect. Bold characters are used for groups of compounds.

Opm: sterretjes aanpassen? * en ** omwisselen om gelijk te trekken met figuur 2?

Supplemental table 2: Experimental effects observed for the protein bound compounds

	Inflam-mation	*CVD*	*CKD-MBD*	*Fibrosis*	*Infection*	*Insulin resistance*	*Metabolic function*	*PEW*	*Neuro-toxicity*	*Thrombo-genicity*	*Hemato-logy*	**Sum**
**AGEs***												7
**AOPPs***												3
CMPF												2
p-Cresyl sulfate												7
p-Cresyl glucuronide												2
Hippuric acid												3
p-OH hippurate												4
o-OH hippurate												2
Homocysteine												3
Indoxyl sulfate												6
Indole acetic acid												5
Indoxyl glucuronide												1
**Kynurenines***												5
Phenyl sulfate												1
Phenyl acetic acid												5
Quinolinic acid												3
Sum	11	9	5	8	2	4	11	0	4	4	1	

Metabolic function includes tubular function; fibrosis includes progression of CKD; CVD: Cardiovascular Disease; CKD-MBD: Chronic Kidney Disease – Metabolic Bone Disease; PEW: Protein Energy Wasting; AGEs: Advanced Glycation End Products, AOPPs: Advanced Oxidation protein Products; CMPF: Carboxy Methyl Propyl Furanpropionic Acid; p-OH hippurate: p-hydroxyhippurate; o-OH hippurate: o-hydroxyhippurate; *: AGEs, AOPPs en kynurenines considered as one group. Colored for demonstrated effect. Bold characters are used for groups of compounds.

Supplemental table 3: Experimental effects observed for the middle molecules

	Inflam-mation	*CVD*	*CKD-MBD*	*Fibrosis*	*Infection*	*Insulin resistance*	*Metabolic function*	*PEW*	*Neuro-toxicity*	*Thrombo-genicity*	*Hemato-logy*	*Sum*
Adrenomedullin												1
Adiponectin												0
Angiogenin												3
ANP												1
β_2-microglobulin												6
β-endorphin												1
β-lipotropin												1
Cholecystokinin												0
Complement factor D												3
Complement factor Ba												1
Cystatin C												1
Interleukin-1β												3
Interleukin-18												3
Interleukin-6												3
TNF-α												3
Interleukin-8												3
Interleukin-10												1
Endothelin												3
FGF-23												3
Ghrelin												5
Glomerulopressin												0
**IgLC***												3
**Lipids & lipoproteins*,****												3
Leptin												5
MCSF												3
Methionine-enkephalin												2

Neuropeptide Y												3
Orexin A												1
Parathyroid hormone												5
Pentraxin-3												4
Peptide YY												2
Prolactin												1
Resistin												2
Retinol binding protein												4
Visfatin												0
Sum	16	21	4	12	5	4	7	4	6	2	2	

Metabolic function includes tubular function; fibrosis includes progression of CKD; CVD: Cardiovascular Disease; CKD-MBD: Chronic Kidney Disease – Metabolic Bone Disease; PEW: Protein Energy Wasting; ANP: atrial natriuretic peptide, TNF-α: Tumor Necrosis Factor-α, IgLC: Immunoglobulin Light Chains; MCSF: Macrophage Colony Stimulating Factor; *: Immunoglobulin Light Chains and lipids and lipoproteins (modified) considered as one group; **: modified – post-translational. Colored for demonstrated effect. Bold characters are used for groups of compounds.
